# Rational combinatorial targeting by adapter CAR-T-cells (AdCAR-T) prevents antigen escape in acute myeloid leukemia

**DOI:** 10.1038/s41375-024-02351-2

**Published:** 2024-08-03

**Authors:** Daniel Atar, Lara Ruoff, Anna-Sophia Mast, Simon Krost, Moustafa Moustafa-Oglou, Sophia Scheuermann, Beate Kristmann, Maximilian Feige, Aysegül Canak, Kathrin Wolsing, Lennart Schlager, Karin Schilbach, Latifa Zekri, Martin Ebinger, Daniel Nixdorf, Marion Subklewe, Johannes Schulte, Claudia Lengerke, Irmela Jeremias, Niels Werchau, Joerg Mittelstaet, Peter Lang, Rupert Handgretinger, Patrick Schlegel, Christian M. Seitz

**Affiliations:** 1 Department of General Pediatrics, Hematology and Oncology, University Children’s Hospital, Tuebingen, Germany; 2 Excellence cluster iFIT (EXC 2180) “Image-Guided and Functionally Instructed Tumor Therapies”, Tübingen, Germany; 3 German Cancer Consortium (DKTK) and German Cancer Research Center (DKFZ), Partner site Tübingen, Tübingen, Germany; 4 Clinical Collaboration Unit Translational Immunology, German Cancer Consortium (DKTK), Department of Internal Medicine, University Hospital Tübingen, Tübingen, Germany; 5 Department of Immunology, IFIZ Institute for Cell Biology, Eberhard Karls University of Tübingen, Tübingen, Germany; 6 Department of Medicine III, University Hospital, LMU, Munich, Germany; 7 Laboratory for Translational Cancer Immunology, LMU Gene Center, Munich, Germany; 8 Department of Internal Medicine II, Hematology, Oncology, Clinical Immunology, and Rheumatology, University Hospital Tübingen, Tübingen, Germany; 9 Research Unit Apoptosis in Hematopoietic Stem Cells, Helmholtz Center Munich, Munich, Germany; 10 German Cancer Consortium (DKTK), partner site Munich, Munich, Germany; 11 Department of Pediatrics, Dr. Von Hauner Children’s Hospital, LMU University Hospital, LMU Munich, Munich, Germany; 12 R&D Department, Miltenyi Biotec B.V. & CO. KG, Bergisch Gladbach, Germany; 13 School of Medical Sciences, Faculty of Medicine and Health, University of Sydney, Sydney, NSW Australia; 14 Hopp-Children’s Cancer Center Heidelberg (KiTZ), Heidelberg, Germany; 15 Department of Pediatric Oncology, Hematology, and Immunology, Heidelberg University Hospital, Heidelberg, Germany

**Keywords:** Acute myeloid leukaemia, Acute myeloid leukaemia, Cancer immunotherapy, Cancer immunotherapy, Preclinical research

## Abstract

Targeting AML by chimeric antigen receptor T-cells (CAR-T) is challenging due to the promiscuous expression of AML-associated antigens in healthy hematopoiesis and high degree of inter- and intratumoral heterogeneity. Here, we present single-cell expression data of AML-associated antigens in 30 primary pediatric AML samples. We identified CD33, CD38, CD371, IL1RAP and CD123 as the most frequently expressed. Notably, high variability was observed not only across the different patient samples but also among leukemic cells of the same patient suggesting the necessity of multiplexed targeting approaches. To address this need, we utilized our modular Adapter CAR (AdCAR) platform, enabling precise qualitative and quantitative control over CAR-T-cell function. We show highly efficient and target-specific activity for newly generated adapter molecules (AMs) against CD33, CD38, CD123, CD135 and CD371, both in vitro and in vivo. We reveal that inherent intratumoral heterogeneity in antigen expression translates into antigen escape and therapy failure to monotargeted CAR-T therapy. Further, we demonstrate in PDX models that rational combinatorial targeting by AdCAR-T-cells can cure heterogenic disease. In conclusion, we elucidate the clinical relevance of heterogeneity in antigen expression in pediatric AML and present a novel concept for precision immunotherapy by combinatorial targeting utilizing the AdCAR platform.

## Introduction

Chimeric antigen receptor-expressing T-cells (CAR-T) have transformed the therapeutic landscape in B-phenotypic malignancies, demonstrating clinical efficacy in lymphoblastic leukemia, lymphoma and myeloma [1–4]. In contrast, no clinical breakthrough has been achieved yet in AML and other myeloid neoplasia. Multiple factors contribute to the complexity of targeting AML by CAR-T-cells. AML consists of vastly phenotypically and functionally heterogeneous blasts organized in a hierarchical system. Less differentiated stem-like cells, referred to as leukemic stem cells (LSCs), are responsible for disease initiation, relapse and therapy resistance [5, 6], while more mature bulk populations further contribute to intratumoral heterogeneity and plasticity [7]. CD33 (SIGLEC3), CD123 (IL3RA) and CD371 (CLL1 or CLEC12A) have been identified as potential candidates for CAR-T targeting but unfortunately show highly promiscuous expression profiles with strong overlap in healthy counterpart hematopoietic stem and progenitor cells (HSPCs) [8, 9]. While B-cell aplasia in the context of CD19-targeted CAR-T-cells is clinically manageable, direct targeting of HSPCs leads to profound myeloablation associated with severe risks for infectious complications that cannot be equally resolved by currently available supportive measures. Thus, tight regulation of CAR-T-cell function is needed to allow the regeneration of healthy myelopoiesis after targeting AML with CAR-T-cells. Furthermore, due to phenotypic heterogeneity [10], it appears unlikely that AML can be cured by targeting a single antigen. In addition to the already documented escape mechanisms mediated by antigen downregulation, as observed after CD19 targeting in B-lymphoid malignancies, AML relapses are prone to occur from outgrowth of preexisting antigen-negative subclones.

To address these challenges, we recently developed the adapter CAR platform (AdCAR) [11] to provide a highly flexible platform for qualitatively, quantitatively and temporally controlled antigen targeting by T and other effector cells. AdCAR is directed against a biotin tag in the context of a specific linker structure, referred to as a linker-label epitope (LLE), that can be conjugated to any kind of binding molecule (e.g., monoclonal antibodies (mAbs), mAb fragments, natural or synthetic ligands), referred to as an adapter molecule (AM). AdCAR-expressing effector cells are redirected to a surface antigen and activated against the target cell only in the presence of antigen-specific AMs. In addition to tight control of effector cell function, this system allows both simultaneous and sequential targeting of multiple antigens by AdCAR-expressing effector cells, thus paving the road to personalized combinatorial targeting approaches [11–18]. In the present study, we utilized the AdCAR platform to address major limitations of conventional CAR approaches in AML, with a special focus on pediatric AML. We provide a comprehensive overview of both inter- and intratumoral heterogeneity in target antigen expression in pediatric AML and identify preexisting antigen-negative or low subpopulations prone to antigen escape. Importantly, we used a patient-derived xenograft model to show that AdCAR-T-cells targeting multiple antigens (*n* = 3) can induce complete and sustainable remission in heterogeneous AML, thus providing proof-of-concept data demonstrating the vast superiority of the AdCAR-T strategy over single targeted approaches.

## Methods

### Generation of novel AMs, mAb production and biotin conjugation

IgG1 against CD33, CD38, CD123, CD135, and CD371 were generated using the ExpiCHO™ expression system (Thermo Fisher). To this end, VH and LH sequences for the respective mAbs were cloned on an Fc-attenuated (L234A/L235A (LALA)) IgG1 backbone in a pcDNA™3.1 ^(+)^ mammalian expression vector. Transfection was performed according to the manufacturer’s instructions using the high titer protocol. Antibodies in the clarified and filtered supernatant were purified using protein A GraviTrap columns (Cytiva) followed by desalting with PD10 Sephadex G25 columns (Cytiva). Purity was analyzed by SDS gel analysis. Biotin conjugation of purified antibodies was performed using a three-fold molar excess of biotin-LC-LC-NHS (Thermo Fisher), followed by separation of the antibody/label mix on a Sephadex G25 column (Cytiva). Successful conjugation was confirmed by flow cytometry on cell lines expressing the target antigen and secondary staining with a fluorophore-conjugated antibiotin antibody (Miltenyi Biotec) and functional assays.

### Isolation of human primary T-cells and CAR-T generation

Peripheral blood mononuclear cells (PBMCs) were isolated from whole blood samples acquired from healthy volunteer donors at the University Children’s Hospital Tübingen (approved by the Institutional Ethical Review Board 761/2015BO2) by Ficoll-Paque density gradient (Biocoll, Biochrom). T-cells were isolated by magnetic separation using anti-CD4/8 microbeads (Miltenyi Biotec), stimulated with TransAct^TM^ (anti-CD3/28 agonistic signal) (Miltenyi Biotec) and cultivated in TexMACS media supplemented with 10 ng/mL IL7 and 5 ng/mL IL15 (Miltenyi Biotec) for up to 15 days. Transduction with an MOI of 3 was performed 36 h after stimulation. Transduction efficacy was determined on day +7 by flow cytometry with the AdCAR detection reagent PE (Miltenyi Biotec).

### Immunophenotyping of bone marrow from pediatric AML patients and healthy donors

Patient characteristics are provided in Supplementary Table 1. This study was approved by the Institutional Ethical Review Board (“Ethikkommission der Medizinischen Fakultät der Eberhard-Karl-Universität und am Universitätsklinikum Tübingen” approval number 819/2017BO1, 674/2017BO2) and performed in accordance with the Declaration of Helsinki. Informed consent was obtained from all patients. Patient samples were treated with DNase 1 (Thermo Fischer) in DPBS prior to staining and incubated with the FcR blocking reagent human (Miltenyi Biotec). A list of antibodies, fluorochromes, dilution factors and live/dead staining used in this study is provided in the supplementary materials. Samples were acquired and unmixed on a Cytek Aurora (CytekBiosciences) 5-laser spectral flow cytometer using the “Cytek Assay Settings” in SpectroFlo® V3.0.3 software and further analyzed using FlowJo 10.8 software. Antigen positivity was defined by fluorescence minus one (FMO) controls. Uniform manifold approximation and projection were calculated using FlowJo 10.8 software and Plugin “UMAP” (arXiv:1802.03426).

### Flow cytometry-based cytotoxicity assays

Wild-type, CD33KO, CD38KO, and CD33/CD38KO cells were mixed at a 1:1:1:1 ratio using 1.5 × 10^5^ cells per variant (total cell number 6 × 10^5^). AdCAR-T-cells were added at an E:T ratio of 1:1. AMs only or combinations thereof were added to a final concentration of 10 ng/mL as indicated. Tubes were incubated for the indicated time at 37 °C, 95% humidity, and 5% CO2. At the experimental endpoint, cells were stained for CD33, CD38, CD123, CD135, CD371, CD45 and CD3. 7-AAD was added to exclude dead cells. A list of antibodies, fluorochromes and dilution factors is provided in the supplementary materials. Samples were acquired with a BD FACSCanto™ II flow cytometer (BD Biosciences) using BD FACSDiva™ software and further analyzed using FlowJo 10.8 software. Uniform manifold approximation and projection were calculated using FlowJo 10.8 software and Plugin “UMAP” (arXiv:1802.03426).

### Animals, PDX models, and in vivo studies

For all experiments, 6- to 8-week-old female NOD. Cg-*Prkdc*^*scid*^ *Il2rg*^*tm1Wjl*^/SzJ (NSG) mice from Charles River Laboratories were used. The general health status of all animals was monitored daily. All experiments were performed according to the guidelines of the Federation of European Laboratory Animal Science Associations (FELASA) in the animal husbandry facilities of the University Clinic Tübingen. For PDX generation, mono nuclear cells were isolated by Ficoll-Paque density gradient (Biocoll, Biochrom) from fresh bone marrow of pediatric AML patients and transplanted immediately via teil vein injection into NSG mice. After engraftment, AML blasts were isolated from murine bone marrow by magnetic separation using anti-human CD45 microbeads (Miltenyi Biotec), transduced with a lentiviral vector encoding firefly luciferase and CD19t, and retransplanted into a mouse. After the second mouse passage, transduced AML blasts were enriched by magnetic separation using anti-human CD19 microbeads (Miltenyi Biotec), analyzed, and cryopreserved. For in vivo studies, NSG mice were engrafted with either 1 × 10^6^ U937 cells on day −4 or 1 × 10^6^ PDX cells on day −3 via tail vein injection. A total of 5 × 10^6^ CAR-T-cells were injected intravenously on day 0. Twice a week, 45 µg of the indicated AMs or combinations thereof were injected subcutaneously (*s.c*.), beginning on day 0. Tumor growth was monitored by bioluminescence imaging (BLI). A detailed description of the performed in vivo studies is provided in the supplementary materials.

### Statistical analysis

Statistical analysis was performed using two-way ANOVA and Tukey’s multiple comparison test. Ns, not significant, *p* ≤ 0.0332 (*), *p* ≤ 0.0021 (**), *p* ≤ 0.0002 (***), *p* ≤ 0.0001 (****). The full table of the respective statistical analysis is provided in Additional file 1.

## Results

### Target antigens are heterogeneously expressed in pediatric AML

**Fig. 1:**
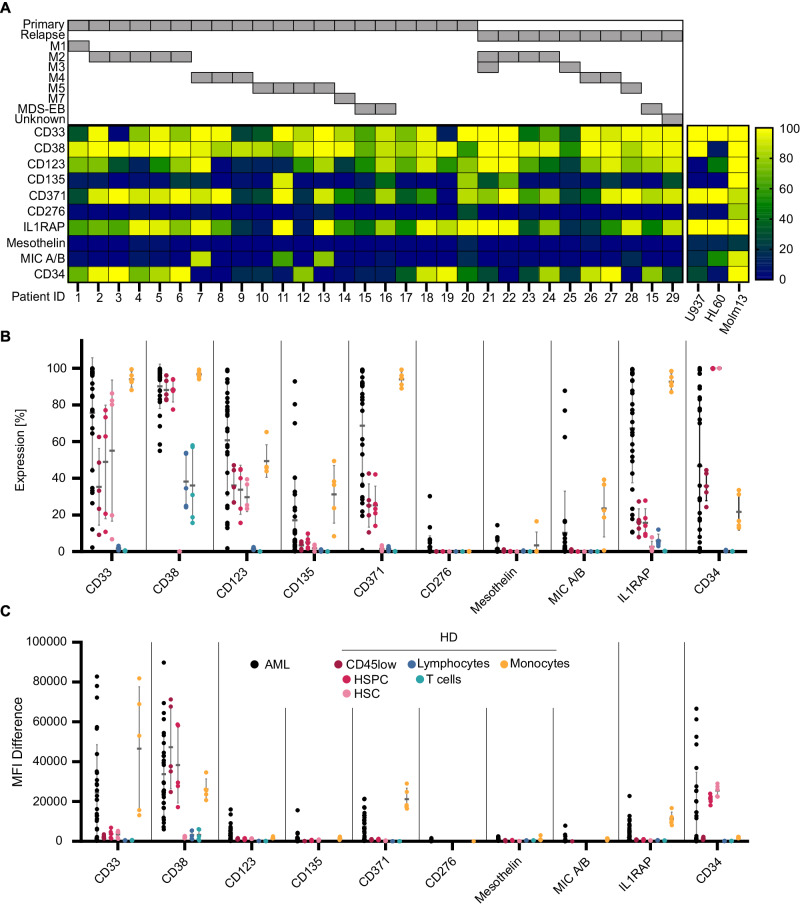
Expression of target antigens in pediatric AML and healthy bone marrow. **A** Heatmap visualizing the percentage of leukemic blasts, defined as CD45^dim^ cells within the BM of pediatric AML patients (*n* = 30), expressing the target antigens CD33, CD38, CD123, CD135, CD371, CD276, IL1RAP, Mesothelin, or MIC A/B as well as CD34, analyzed by flow cytometry. Patients are grouped by primary disease vs. relapse as well as FAB classification/MDS-EB. Target expression of the used AML cell lines are visualized as heatmap next to patient data (right). Comparison of target antigen expression between AML blasts and major cell populations (defined as follows: HSC (CD45^dim/^CD34^high^/CD38^low^), HSPC (CD45^dim/^CD34^high^/CD38^high^), lymphocytes (CD45^high^/SSC^low^), T cells (CD45^high^/SSC^low^/CD3^high^), and monocytes (CD45^high^/SSC^median^)) in bone marrow from healthy donors (*n* = 5). In **B** the percentage of antigen-positive cells is provided; in **C** the mean fluorescence intensity difference (MFID). Each data point represents an individual patient or heathy donor, and horizontal lines represent the mean value ± standard deviation (SD).

To study target antigen expression in pediatric AML at single-cell resolution, a representative cohort of primary AML samples (*n* = 30) at diagnosis (*n* = 20) and relapse (*n* = 10), including one matched diagnoses/relapse sample, was analyzed by multicolor flow cytometry for CD45, CD34, and CD3 as well as CD33, CD38, CD123, CD135, CD371, CD276, IL1RAP, mesothelin and MICA/B as potential target antigens. An overview of antigen expression patterns on AML blasts and consents is illustrated in Fig. 1A. Patient characteristics are provided in Supplementary Table 1, the gating strategy in Supplementary Fig. 1A. In line with a previous report [19], only a minority of samples (3/30) showed CD34^high^/D38^low^ subpopulations reported as LSC enriched in adult AML, suggesting important differences between pediatric and adult patients. In the present cohort, CD33 and CD38 were expressed most frequently in the majority of blasts (23/30 and 30/30 with >50% positive blasts) and at the highest MFI difference (MFID), followed by IL1RAP (22/30), CD123 (21/30), CD371 (21/30), CD135 (3/30) and MICA/B (3/30) (Fig. 1B and C), with no documented difference between samples collected at initial diagnosis versus relapse. In contrast, CD276 and Mesothelin showed no relevant expression. Antigen expression was further compared to expression on healthy blood cell subsets. The gating strategy is provided in Supplementary Fig. 1B. None of the AML-associated antigens were found to be exclusively expressed on AML blasts (Fig. 1B and C). Healthy HSPCs further showed expression of CD33, CD123, CD371 and IL1RAP, although at a lower frequency and MFID than leukemic blasts. In contrast, CD135, CD276, Mesothelin and MICA/B were not expressed on healthy blood cells. CD38 is known to be absent on healthy HSCs but expressed on HSPCs at comparable levels to AML blasts, both in frequency and MFID. Monocytes, one of the most frequent myeloid cell populations, express CD33, CD38, CD123, CD135, CD371 and IL1RAP at high frequency and MFID. CD38 is further expressed at lower levels on lymphocytes and T-cells. Both CD38 and CD33 were found to be expressed on CD3/CD28 activated T-cells (Supplementary Fig. 1D). In conclusion, our analyses reveal no ideal target antigen, which is consistently present on AML blasts but absent on healthy blood cells. High intertumoral heterogeneity in pediatric AML will require multiple target antigens to address the disease as a whole.

### Novel adapter molecules demonstrate antigen-specific activity against AML cell lines

**Fig. 2:**
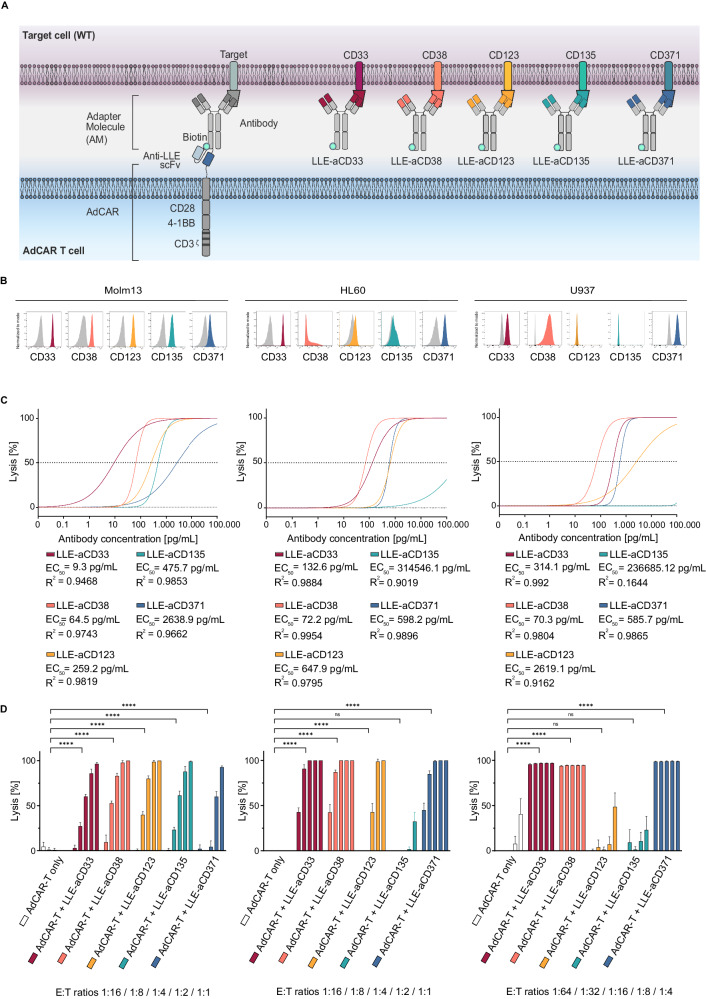
Design and in vitro evaluation of novel AML-targeted AMs. **A** Schematic illustration of the AdCAR-T system. AdCAR-T cells are directed to AML-associated target antigens via LLE-conjugated mAbs (LLEa-CD33, LLEa-CD38, LLEa-CD123, LLEa-CD135, and LLEa-CD371), referred to as AMs. **B** Histograms of target antigen expression (CD33, CD38, CD123, CD135 and CD371) on AML cell lines (Molm13, HL60 and U937) stained with the indicated AM and secondary anti-LLE mAb, analyzed by flow cytometry. **C** Cytotoxicity of AdCAR-T against the indicated luciferase-expressing AML cell lines as determined by LCA after 48 h at an E:T of 1:1 (Molm13, HL60) or 1:4 (U937), mediated by increasing concentrations, logarithmic titration steps from 0.1 pg/mL to 100 ng/mL, of the indicated AMs. EC_50_ values are provided for each AM. **D** Cytotoxicity of AdCAR-T cells against the indicated AML cell lines as determined by LCA after 48 h at fixed AM concentrations (10 ng/mL) and indicated E:T ratios. AdCAR-T cells in the absence of AM served as a negative control. Data shown in C and D represent the mean ± SD of (*n* = 6). Data shown in C were transformed by taking the logarithm of the x values and then fitted by nonlinear regression. Statistical analysis was performed using two-way ANOVA and Tukey’s multiple comparison test. ns, not significant. *p ≤ 0.0332 **p ≤ 0.021. ***p ≤ 0.002. ****p ≤ 0.0001. The full table of the statistical analysis is provided in Additional file 1.

To address the intertumoral heterogeneity in AML, a panel of AMs was generated, targeting the AML-associated antigens CD33, CD38, CD123, CD135 and CD371 (Fig. 2A). Targets were prioritized based on our expression data, published pre-clinical and clinical results as well as the availability of validated mAbs. To this end, VH and LH sequences for the mAbs CD33 (hP67.6, gemtuzumab), CD38 (daratumumab), CD123 (7G3, WO2016201065A1), CD135 (4G8 FLT3, EP3623383A1), and CD371 (h6E7.L4H1e. A54, EP3191520A1) were cloned into an Fc-attenuated (L234A/L235A (LALA)) IgG1 backbone [20] to minimize Fc-receptor-mediated ADCC and AdCAR-T-independent effects. After synthesis in CHO cells and purification, mAbs were LLE-conjugated, now referred to as LLE-“target-specificity”mAb (Supplementary Fig. 2). Specific target binding and LLE conjugation were verified by flow cytometry using a fluorochrome-labeled anti-LLE mAb (Fig. 2B). For functional testing of AMs with AdCAR-T-cells, three AML cell lines showing different antigen expression profiles were utilized: MOLM13 (CD33^high^, CD38^high^, CD123^high^, CD135^high^, CD371^low^), HL60 (CD33^high^, CD38^low^, CD123^low^, CD135^low^, CD371^high^) and U937 (CD33^high^, CD38^high^, CD123^low^, CD135^low^, CD371^high^) (Fig. 2B). AM titration experiments demonstrated highly specific, target antigen-dependent lysis of AML cells by AdCAR-T-cells at very low AM concentrations (EC_50_ range 9.3–647.9 pg/mL for highly expressed antigens) (Fig. 2C). Efficient target cell lysis was observed even at a low effector to target (E:T) ratio (Fig. 2D). In contrast, no specific lysis was found for AdCAR-T-cells only or if the target antigen was not expressed (Fig. 2D). Taken together, these data demonstrate that the AdCAR-T platform can easily be adapted to a heterogeneous disease such as AML, allowing flexible targeting of multiple different antigens.

### AdCAR-T-cells demonstrate specific in vivo activity, allowing individualized targeting

**Fig. 3:**
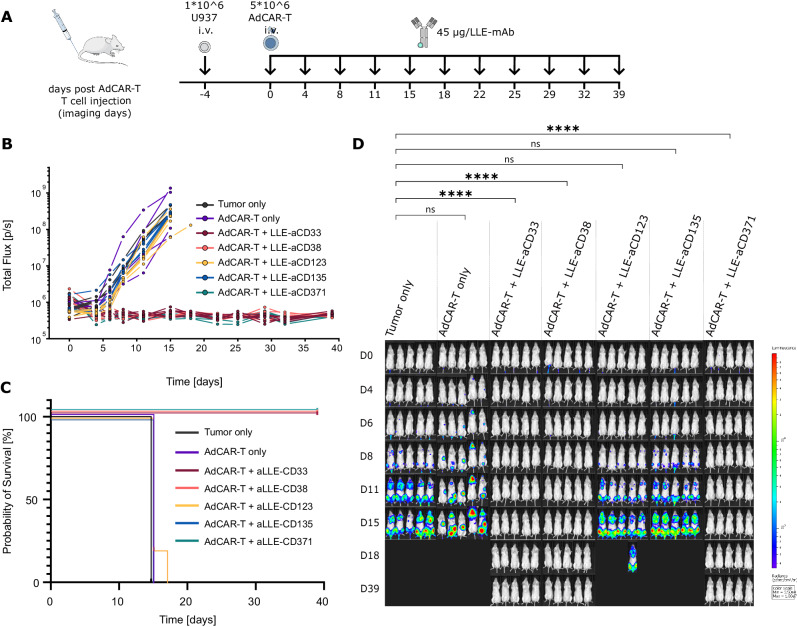
In vivo validation of target antigen-specific activity of AdCAR-T cells. **A** Schematic depiction of the in vivo experiment: NSG mice were engrafted with 1 × 10^6^ U937^luc/CD19t^ (CD33^high^, CD38^high^, CD123^low^, CD135^low^, CD371^high^) on day −4 via tail vein injection (i.v.). A total of 5 × 10^6^ AdCAR-T cells were injected i.v. on day 0. Forty-five micrograms of the indicated AM (LLE-aCD33, LLE-aCD38, LLE-aCD123, LLE-aCD135 or LLE-aCD371) was injected subcutaneously (s.c.) twice a week starting on day 0. Untreated mice (tumor only) served as a negative control (*n* = 5 per group). Tumor load was monitored by BLI. Mice were sacrificed when they reached the endpoint criteria. **B** Total flux [photons/second] of in vivo bioluminescence blotted over time for individual animals. **C** Kaplan‒Meier curves for reaching endpoint criteria. **D** BLI images at the indicated time points (exposure time 10 sec.). Statistical analysis was performed using two-way ANOVA and Tukey’s multiple comparison test. ns, not significant. *p ≤ 0.0332 **p ≤ 0.021. ***p ≤ 0.002. ****p ≤ 0.0001. The full table of the statistical analysis is provided in Additional file 1.

High intertumoral heterogeneity will require personalized targeting approaches based on patient individual antigen expression profiles. After demonstrating specific in vitro activity for different AMs, AdCAR-T-cells were tested in vivo to demonstrate the feasibility of individualized targeting. To this end, NSG mice were engrafted with U973 AML cells (CD33^high^, CD38^high^, CD123^low^, CD135^low^, CD371^high^) and treated with AdCAR-T-cells plus AMs targeting CD33, CD38, CD123, CD135 or CD371 (Fig. 3A). Underscoring the potency of AdCAR-T-cells, treatment with AdCAR-T-cells in combination with AMs targeting CD33, CD38 or CD371 induced lasting remission, indicating complete AML cell eradication. In contrast, untreated control groups or treatment with AdCAR-T-cells in combination with AMs in samples with low CD123 or CD135 expression showed no similar effects (Fig. 3A–D). Together, these data demonstrate the specific in vivo activity of AdCAR-T-cells for personalized multiantigen targeting approaches. Of note, although expressed on a subpopulation of activated T-cells, targeting CD38 did not impair the antileukemic activity of AdCAR-T-cells in vivo.

### Primary AMLs show a high degree of intratumoral heterogeneity

**Fig. 4:**
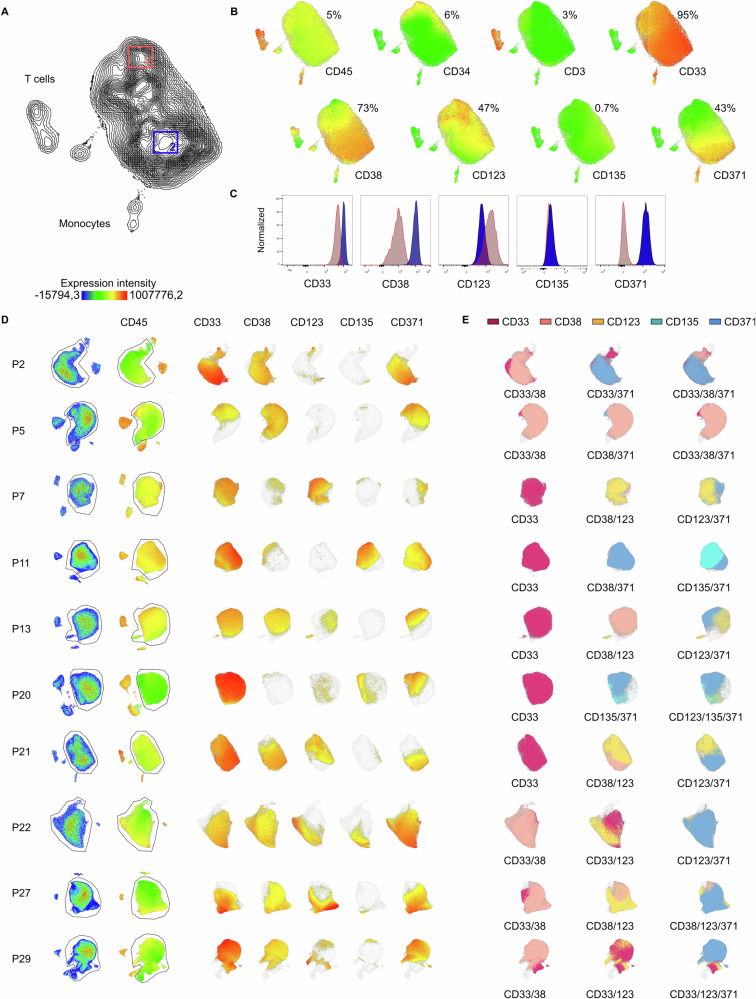
Intratumoral heterogeneity in target antigen expression in pediatric AML. **A** UMAP based on the expression of CD45, CD3, CD34, CD33, CD38, CD123, CD135 and CD371 as well as FSC and SSC signals, as determined by flow cytometry, of an exemplary pediatric AML bone marrow sample (P21). **B** Color-coded intensity of CD45, CD3, CD34, CD33, CD38, CD123, CD135 and CD371 expression, with each dot representing one cell. **C** To highlight intratumoral heterogeneity in target antigen expression, expression of CD33, CD38, CD123, CD135 and CD371, plotted as histograms, in two different areas of the AML blast population, gated and labeled as 1 (LSC-like) and 2 (bulk). **D** UMAPs of AML bone marrow samples (P2, P5, P7, P11, P13, P20, P21, P22, P27, and P29); the left row shows bulk bone marrow as a pseudo color dot plot, and the second row shows CD45 expression and AML blast gates, followed by the expression of target antigens CD33, CD38, CD123, CD135 and CD371. **E** Suggestions of possible target combinations to cover most leukemic blasts.

To further explore antigen heterogeneity in pediatric AML, 10 primary AML samples (mean percentage of blasts 85% (76–92%), mean number of analyzed AML blasts per patient: 75.700 (28.000–143.000)) were analyzed by dimensionality reduction using uniform manifold approximation and projection (UMAP) based on the expression of CD45, CD3, CD34, CD33, CD38, CD123, CD135 and CD371 as well as FSC and SSC signals. As demonstrated for healthy BM (Supplementary Fig. 3), unsupervised clustering accurately differentiates the main cell populations, further validating specific coexpression patterns as described above. In contrast, BMs from AML patients were dominated by leukemic blast clusters (Fig. 4A and Supplementary Fig. 3). Importantly, antigen expression was highly heterogeneous within the analyzed leukemic cell clusters. For example, in the sample shown in Fig. 4A, CD33 was expressed on the majority of blasts, while CD123 expression was restricted to an LSC-like (CD34^pos^/CD38^neg^) subpopulation, and CD38 and CD371 expression was documented on subsets of the bulk population (Fig. 4B). Significant intratumoral heterogeneity was confirmed in all analyzed samples (Fig. 4C). In 6 out of 10 samples, combinations of at least two target antigens were identified as needed to allow targeting of >90% blasts (Fig. 4E). A comprehensive overview of coexpression is provided in Additional file 2. Together, these data strongly suggest that mono-antigen targeting is insufficient to treat AML.

### Multiplex targeting by AdCAR-T-cells can successfully address intratumoral heterogeneity

**Fig. 5:**
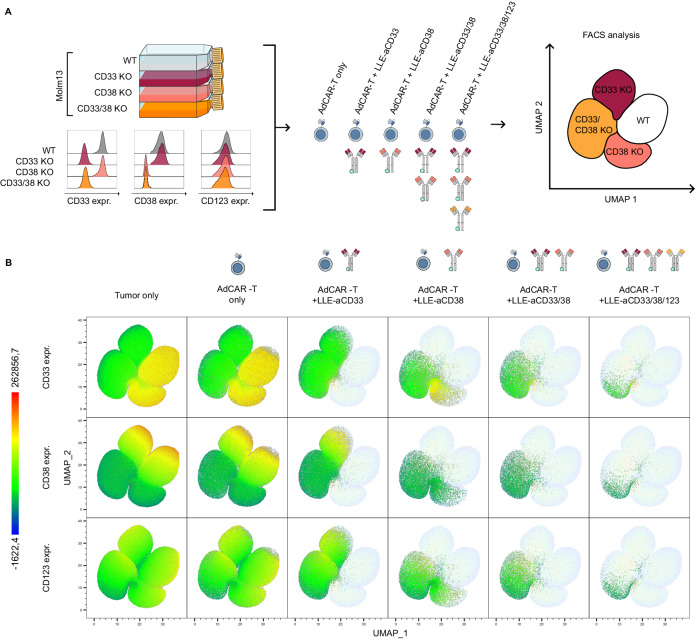
In vitro evaluation of multiplex targeting by AdCAR-T. **A** Schematic depiction of the flow cytometry-based cytotoxicity assay: Molm13 wild type (WT), Molm13 CD33KO, Molm13 CD38KO, and Molm13 CD33/CD38 KO were mixed at a 1:1:1:1 ratio. The expression of CD33, CD38, and CD123 in individual cell populations is shown on the lower left. AdCAR-T cells were added at an E:T ratio of 1:1. LLE-aCD33, LLE-aCD38, or LLE-aCD123 as well as combinations thereof were added to reach a final concentration of 10 ng/mL. **B** UMAP representing batched surviving target cells after 48 h of incubation of all conditions (*n* = 3 each condition), based on the expression of CD33, CD38 and CD123, as determined by flow cytometry. From left to right, viable target cells for the indicated conditions plotted in color. From top to bottom, color-coded expression of CD33, CD38, and CD123.

To model intratumoral heterogeneity in a controlled fashion, knockout variants for CD33, CD38 or both CD33 and CD38 were generated in AML cell lines using CRISPR/Cas9 (Fig. 5A). Validating the specificity of AM-mediated targeting, knockout of target antigens completely abrogated AdCAR-T lysis, demonstrating the antigen specificity of this targeting approach. To mimic antigen heterogeneity, AdCAR-T-cells were cocultured with a 1:1:1:1 mixture of wild-type and knockout AML cells (MOLM13^WT^, MOLM13^CD33KO^, MOLM13^CD38KO^ and MOLM13^CD33/CD38KO)^ (Fig. 5A and B). No specific lysis was found in the absence of AMs (Fig. 5B*left*). The addition of single AMs, either against CD33, CD38 or a combination thereof, specifically led to the elimination of antigen-positive target cells (Fig. 5B). The addition of a third AM targeting CD123 further reduced the CD33- and CD38-negative target population (Fig. 5B*right*). Even triple targeting, CD33, CD38 and CD123, by AdCAR-T-cells at a low E:T ratio of 1:4 resulted in the selection of preexisting or forced CD123^low^ target cells (Fig. 5B). This was not observed at higher E:T ratios, indicating target-independent bystander killing (Fig. 2D). Findings were validated in the U937 cell line, combining AMs against CD33, CD38 and CD371 (Supplementary Fig. 4). Together, this set of data demonstrates the feasibility of simultaneous targeting of multiple (*n* = 3) antigens by AdCAR-T-cells to address intratumoral heterogeneity in AML.

### Single antigen targeting of PDX AML results in rapid antigen-negative relapse

**Fig. 6:**
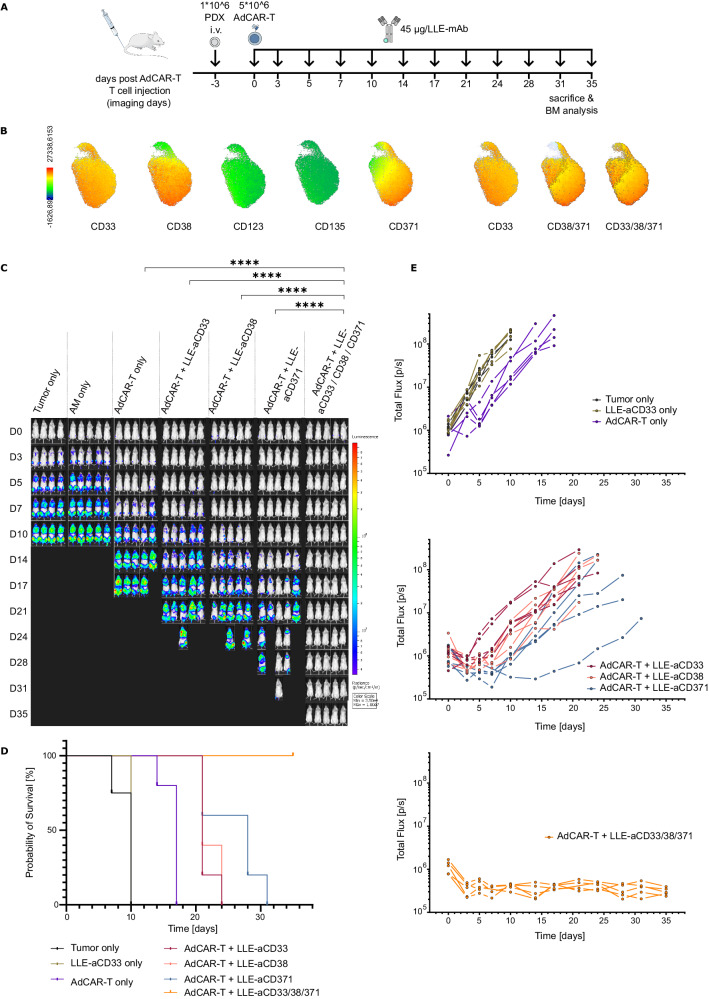
In vivo validation of multiplex targeting by AdCAR-T cells in a PDX model. **A** Schematic depiction of the in vivo experiment: NSG mice were engrafted with 1 × 10^6^ PDX ^luc/CD19t^ (P21) cells on day −3 via tail vein injection (i.v.). A total of 5 × 10^6^ AdCAR-T cells were injected i.v. on day 0. A total of 45 µg of the indicated AM (LLE-aCD33, LLE-aCD38, or LLE-aCD371) or a combination thereof was injected subcutaneously (s.c.) twice a week starting on day 0. Untreated mice (tumor only), mice injected with PBS instead of AM (AdCAR-T only), and mice injected with LLE-aCD33, LLE-aCD38 and LLE-aCD371 but not AdCAR-T (AM only) served as negative controls (*n* = 5 per group, *n* = 4 in tumor only). Tumor load was monitored by BLI. Mice were sacrificed when they reached the endpoint criteria. **B** UMAP based on the expression of CD45, CD3, CD34, CD33, CD38, CD123, CD135 and CD371 as well as FSC and SSC signals, as determined by flow cytometry, of PDX cells prior to injection, expression of CD33, CD38, CD123, CD135 and CD371 on AML blasts (left) and suggestions for combinatorial targeting (right). **C** BLI images at the indicated time points (exposure time 10 s). **D** Total flux [photons/second] of in vivo bioluminescence blotted over time for individual animals. **E** Kaplan‒Meier curves for reaching endpoint criteria. Statistical analysis was performed using two-way ANOVA and Tukey’s multiple comparison test. ns, not significant. *p ≤ 0.0332 **p ≤ 0.021. ***p ≤ 0.002. ****p ≤ 0.0001. The full table of the statistical analysis is provided in Additional file 1.

**Fig. 7:**
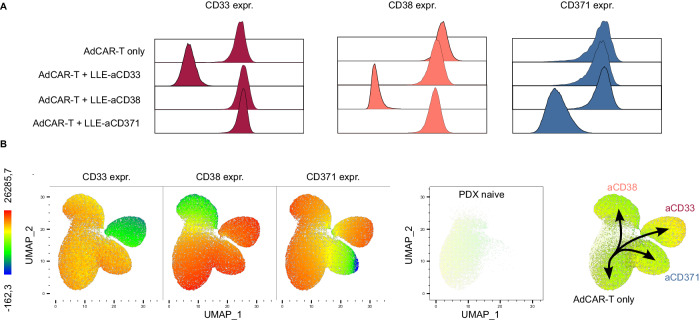
Analysis of resistance mechanisms to mono-targeting. **A** Representative flow cytometry analyses of bone marrow from relapsed mice. Expression of CD33, CD38 and CD371 on viable AML blasts for the indicated groups plotted as histograms. **B** UMAP, representing batched viable AML blasts in bone marrow from tumor-only mice, AdCAR-T-only mice and mice treated with AdCAR-T plus LLE-aCD33, LLE-aCD38, or LLE-aCD123 (*n* = 3 each condition), based on the expression of CD33, CD38 and CD123, as determined by flow cytometry. From left to right, expression of CD33, CD38, and CD371 color-coded, spatial localization of treatment naïve PDX cells ( = tumor only) and pseudotrajectories enforced by mono-targeting.

To evaluate the relevance of intratumoral heterogeneity in primary disease and simulate patient AdCAR-T therapy, PDX models of pediatric AML were treated with AdCAR-T-cells plus single AMs or a rational combination thereof (Fig. 6A). One PDX was derived from patient No. 21 of our target screening cohort (see Fig. 1A and Fig. 4A). After two mouse passages, including lentiviral transduction for luciferase expression and selection, the antigen expression pattern resembled primary disease, in addition to a slight decrease in CD123 (Fig. 6B). Underscoring the therapeutic relevance of intratumoral heterogeneity in antigen expression, single targeting against either CD33, CD38 or CD371 led to rapid disease progression, prolonging the median time to reach end-point criteria in comparison to untreated PDX mice by only 11 for CD33, 11 for CD38 and 18 for CD371 days (Fig. 6C–E). In striking contrast, a rational combination of AMs targeting CD33, CD38 and CD371, expected to address >95% of leukemic blasts (Fig. 6B), led to lasting remission in all mice, indicating the potential of this combinatorial treatment to completely eradicate the disease. Combinatorial targeting resulted in significantly increased CAR-T-cell counts in bone marrow in comparison to AdCAR only and single targeting at the endpoint (Supplementary Fig. 5C). Analyses of resistant disease by flow cytometry, gating strategy for mouse bone marrow provided in Supplementary Fig. 5 reveals a dramatic loss of detectable target antigen expression according to the specificity of the applied AM, suggesting antigen escape by selection of antigen-low or antigen-negative subpopulations (Fig. 7A). Batched UMAPs of untreated PDX cells, AdCAR-T-cells only and resistant disease after mono-targeting with either anti-CD33, anti-CD38 or anti-CD371 AMs clearly separates different therapeutic conditions, revealing trajectories toward antigen-low or antigen-negative disease enforced by a single targeted therapeutic intervention (Fig. 7B). The therapeutic advantage of combinatorial targeting was verified in a second PDX model (Supplementary Fig. 6A-E). Together, these experiments clearly underscores the potential of multiplex targeting by AdCAR-T-cells to cope with antigen escape due to heterogeneous antigen expression.

## Discussion

Targeting AML by CAR-T-cells is notoriously difficult due to shared antigen expression on leukemic cells and healthy HSCs, HSCPs, and cells of the myeloid lineage as well as inter- and intrapatient heterogeneity. In the present study, we evaluated a cohort of primary pediatric AML samples by multicolor flow cytometry to define the landscape of potential target antigens. In line with previous multiomics studies from us and others [8, 9, 19], we demonstrated the expression of the well-described AML-associated antigens CD33 (SIGLEC3), CD38, CD123 (IL3RA) and CD371 (CLL1 or CLEC12A) as well as less frequently CD135 (FLT3) in primary pediatric AML. We further identified IL1RAP as a potential target. IL1RAP expression has been reported in adult-type AML on both bulk leukemic cells and LSCs [21], and IL1RAP-targeted CAR-T-cells are currently being evaluated in a clinical trial (NCT04169022) in adult AML.

However, CD33, CD38, CD123, CD135, CD371 and IL1RAP were coexpressed in healthy HSPCs and myeloid cells, and CD33 and CD38 were coexpressed in subsets of activated T-cells. Previous studies in adult AML have identified CD276, Mesothelin, MICA/B, CD96, LILRB2, CCR1 and CD70 as potential target antigens [8, 22, 23], which could not be confirmed in our pediatric cohort and previously published data. [19] To the best of our knowledge, no AML-exclusive target antigen has been identified in adult or pediatric AML. Targeting promiscuously expressed AML-associated antigens will result in depletion of antigen-positive cell populations, as demonstrated for B-cell-targeted CAR-Ts. Consequently, stringent safety measurements are needed to prevent or react to life-threatening on-target off-tumor activity, such as myeloablation, as demonstrated in preclinical models [24–26].

Conventional CAR-T design does not allow the control of CAR-T activity. Therefore, current clinical approaches applying CAR-T-cells in AML are limited to a bridge-to-transplant setting, terminating CAR-T-cells by lymphodepletion or activation of kill switches [27]. Transient expression of the CAR, for example, by mRNA [28], might improve the safety profile but does not convey lasting disease control. More sophisticated approaches address the safety aspect by gene editing, knocking out the target antigen in hematopoietic stem cells to render them resistant to therapy [29], remote controlled inducible CAR expression [30, 31] or synthetic receptors for logic gating [32, 33].

One elegant way to achieve maximal control of CAR-T activity is to split antigen recognition from CAR-T activation. First introduced by the expression of an Fcγ receptor (CD16) [34] or CD16-derived CAR construct [35] in T-cells to enable antibody-dependent cellular cytotoxicity (ADCC), the concept of “adapter”-mediated CAR-T activation was improved via iteratively in subsequent studies [36–40]. We have recently reported on the development of the AdCAR platform, in which the CAR is directed against biotin in the context of a specific linker structure, referred to as a linker-label epitope (LLE). The LLE-tag can be chemically conjugated to any kind of binding molecule (e.g., mAbs, mAb fragments, natural or synthetic ligands), allowing highly flexible and convenient AM generation [11]. Inherent to the design of all “adapter”-CAR systems is the beneficial safety profile, rendering these approaches a perfect fit for AML. Encouragingly, the first clinical data (NCT04230265) targeting CD123 in adult AML demonstrated rapid recovery of white blood cells and neutrophil counts after termination of AM application. Moreover, induction of complete remission was observed in 2 out of 3 patients, underscoring the feasibility of “adapter”-CAR approaches in AML [41].

The second major obstacle to efficient CAR-T therapy in AML is inter- and intratumoral heterogeneity in target antigen expression. In the present study, we provide data on target antigen expression in a cohort of primary pediatric AML patients at single-cell resolution. We detected vast intertumoral heterogeneity, requiring personalized targeting approaches to cover the disease as a whole. Importantly, we demonstrate as a proof-of-concept that the AdCAR platform can be easily adapted to meet these requirements. We manufactured and functionally validated, both in vitro and in vivo, AMs against 5 clinically relevant AML-associated target antigens, CD33, CD38, CD123, CD135 and CD371. The established workflow allows convenient AM generation by LLE conjugation to rapidly broaden the target repertoire, e.g., by building on clinically tested and available binding molecules.

Selected antigens in this study have been intensively investigated in AML in the context of monoclonal antibodies [42, 43], antibody‒drug conjugates [44], bispecific antibodies [45–47] and CAR-T-cells [24–26, 41, 48–54]. Particularly targeting CD38 by CAR-T-cells has been challenging due to broad expression not only in early hematopoiesis but also in a subset of activated T-cells, described to be functionally associated with a terminal exhausted phenotype [55]. Previous reports described that anti-CD38 CAR-mediated fratricide negatively impacts CAR-T expansion and clinical activity [56, 57]. Unexpectedly, we found CD33 expression on the majority of activated (CAR-)T-cells, which has previously been described for activated NK cells [58]. We were not able to detect impairment of AdCAR-T-cell function by targeting CD33 or CD38, either in vitro or in vivo. Since AdCAR-T-cells are expanded in the absence of AMs, AdCAR-T technology allows to exclude CD33 or CD38 dependent CAR signaling and unintended CAR-T activation during manufacturing. This might result in a beneficial cell product.

Beyond intertumoral heterogeneity, we demonstrate dramatic intratumoral heterogeneity of antigen expression in primary pediatric AML. We show that for the majority of patients, targeting a single AML-associated antigen is insufficient to address all leukemic cells. These findings have major clinical implications since antigen-low or antigen-negative subpopulations are a source for antigen escape and subsequent failure to achieve efficient CAR-T therapy, defined by the elimination of all target-positive cells [59]. Using antigen knockout models, we demonstrated that AdCAR-T-cells can be utilized to simultaneously target multiple AML-associated antigens. Validating our hypothesis, we show in PDX models of pediatric AML, resembling intratumoral heterogeneity of the primary disease, that mono-targeting by CAR-T-cells against CD33, CD38 or CD371 results in rapid antigen escape and disease progression. In contrast, we demonstrate for the first time in vivo that rational combinatorial targeting by AMs against CD33, CD38, and CD371 results in the clearance of inherently heterogeneric disease. These experiments clearly underscore the feasibility of utilizing “adapter”-CAR systems, particularly our AdCAR platform, to target multiple antigens in parallel. Antigen evasion by CAR-T-cells or other targeted immunotherapies is an emerging clinical limitation beyond AML [60]. The application of multiple CAR-T products remains challenging, both from a regulatory and a financial point of view. “Adapter”-CAR systems might provide a neat solution, building on a single CAR-T product redirected against a multiplicity of target antigens.

Together, our findings highlight that successful clinical translation of CAR-T-cells against (pediatric) AML will require stringent safety measures as well as personalized combinatorial targeting approaches. We demonstrate that our AdCAR platform meets these requirements, enabling precise qualitative and quantitative control of CAR-T-cell function as well as multiplex antigen targeting, paving the way toward precision immunotherapy. A phase I/II clinical trial in AML is in preparation.

## Supplementary information

Supplemental Materials

Additional file 1._full statistic

Additional file 2._Antigen double triple positivity

## Data Availability

The datasets generated during and/or analysed during the current study are available from the corresponding author on reasonable request. For original data, please contact christian.seitz@med.uni-heidelberg.de
