# Supplementary material for: Rational combinatorial targeting by adapter CAR-T-cells (AdCAR-T) prevents antigen escape in acute myeloid leukemia
Source: Leukemia. 2024 Aug 3;38(10):2183–95. doi: 10.1038/s41375-024-02351-2 (PMC11436361; doi:10.1038/s41375-024-02351-2)

**Supplementary Material & Methods**

**AdCAR construct and production of LV Vector**

The transfer plasmid encoding the AdCAR was generated by cloning the anti-LLE specific scFv Bio2 4G10 into the coding sequence of a third-generation CAR backbone comprising an hIgG4 hinge, hCD8 transmembrane domain, cytoplasmatic domain of CD28, 4-1BB and CD3ζ.

Lentivirus (LV) was produced in HEK293T cells after transfection with polyethylenimine utilizing a second-generation packaging plasmid, the VSV-G envelope plasmid, and the corresponding transfer plasmid. Virus was pelleted form the supernatant by centrifugation (4500g, 24 h) and resuspended with medium before storage at -80°C.

**Isolation of human primary T cells and transduction**

Peripheral blood mononuclear cells (PBMCs) were isolated from whole blood samples, acquired from healthy volunteer donors at the University Children’s Hospital Tübingen. CD4^+^ and CD8^+^ T cells were isolated simultaneously by magnetic separation using anti-CD4/8 microbeads according to Miltenyi Biotec’s CD8/CD4 Micro Bead protocols (Miltenyi Biotec). Isolated T cells were stimulated with human TransAct^TM^ (1:100) (anti-CD3/28 agonistic signal) (Miltenyi Biotec) and cultivated in TexMACS media supplemented with 10 ng/mL IL7 and 5 ng/mL IL15 (Miltenyi Biotec) for up to 15 days. After 36 h, activated T cells were transduced at a multiplicity of infection (MOI) of 3. Transduced T cells were maintained at 0.5–2 × 10 6 cells/mL in IL7/IL15 supplemented TexMACS® media. On day +7, CAR transduction efficiency were determined by flow cytometry with a AdCAR detection reagent PE (Miltenyi Biotec).

**Antibody production and Biotin conjugation**

IgG1 against CD33, CD38, CD123, CD135, and CD371 were generated using the ExpiCHO™ Expression System (ThermoFisher). To this end, VH and LH sequences for the respective mAbs were cloned on a Fc-attenuated (L234A/L235A (LALA)) IgG1 backbone into a pcDNA™3.1 ^(+)^ Mammalian Expression Vector. For transient transfection of ExpiCHO-S™ cells, 1µg plasmid DNA/mL was used according to the high titer protocol of manufacturer’s instructions. All transfections were performed at 50 mL scale in 250 mL non-baffled flasks (Corning, Cat. 431144). Harvest of antibody was done by centrifugation of supernatant at 5000xg for 30 minutes at 4 °C, followed by filtration through a 0.22 µm filter (Nalgene). Clarified and filtered antibodies were purified using a rProtein A GraviTrap column (Cytiva) followed by desalting with PD10 Sephadex G25 column (Cytiva). Purity was analyzed by SDS Gel analysis.

Biotin conjugation of purified antibodies was performed at 21°C for 1 h in 0,1 M NaHCO3 buffer using 3-fold molar excess of biotin-LC-LC-NHS (Thermo Fisher Scientific, CAS-No. 89889-52-1), followed by separation of the antibody/label mix on a Sephadex G25 column (Cytiva). Protein containing fractions were pooled and concentration was measured by absorption at 280 nm. Successful conjugation was confirmed by flow cytometry on cell lines expressing the target antigen and secondary staining with a fluorophore conjugated anti-biotin antibody and functional assays.

**Generation of luciferase expressing cell lines and cell culturing**

Transfer plasmids, based on a 2nd generation lentiviral vector plasmid, containing luciferase and truncated CD19 (luc/19t) were kindly provided by Irmela Jeremias, Helmholtz Center Munich, Germany. Lentiviral particles (LV) were produced in Lenti-X 293T cells (Takara) after lipofection (Lipofectamin 3000) according to the manufacturer’s instructions utilizing a second-generation packaging plasmid, the VSV-G envelope plasmid, and the corresponding transfer plasmid for expression of the gene of interest. Supernatants containing LV were harvested 24 h after lipofection, concentrated by the Lenti-X concentrator (TaKaRa), and cryopreserved according to the manufacturer’s instructions and stored at -80°C. Cell lines were transduced at a MOI of 3. Transgene expression was confirmed by flow cytometry (Anti-CD19-APC, Miltenyi Biotec) Transduced cells were enriched by magnetic separation using anti-CD19 microbeads according to Miltenyi Biotec’s CD19 Micro Bead protocols (Miltenyi Biotec).

All cell lines (U937, Molm13, HL60) were purchased from ATCC or DSMZ. All cell lines were maintained in RPMI 1640 media supplemented with 10 % heat inactivated fetal bovine serum (FBS) (Thermo Fisher Scientific), 2 mM L-glutamine (Biochrom), 1 mM sodium pyruvate (Biochrom) and 100 units/mL of penicillin and 100 µg/mL of streptomycin (Biochrom), referred to as complete media. All cells were cultured at 37 °C, 5% CO_2_, and regularly tested for mycoplasma.

**Generation of luciferase expressing PDX cells**

The PDX was derived from patient No. 21 of our target screen cohort, see **Figure 1A** and **Figure 4A**. And engrafted in 6-8-week old NOD.Cg-PrkdcSCID Il2rgtm1Wjl/SzJ (NSG) mice of female sex from Charles River Laboratories. Tumor burden was measured by analysis of blood collected from the tail vein. Cells were treated with Red Blood Cell (RBC) Lysis Buffer for 10 min and washed in CliniMACS® PBS/EDTA buffer for staining with fluorophore conjugated antibodies against anti-mouseCD45-PE-Cy7, anti-humanCD45-FITC, and 7-AAD. Samples were acquired on BD FACSCanto™ II flow cytometer in BD FACSDiva™ software and further analyzed using FlowJo 10.8 software. PDX AML cells were freshly isolated from mouse spleen or BM and re-suspended in RPMI-Medium (Life Technologies) supplemented with 20 % fetal calf serum (Biochrom), 5 % L-Glutamin, 1 % Penicillin/Streptomycin, 0.6 % mixture of rh insulin/human transferrin/sodium selenite (Life Technologies), 1 mM sodium pyruvate, and 50 μM 1-thioglycerole (Sigma-Aldrich). 1 x 10^6^ cells in 1 ml medium were transferred to a cell culture plate and were transduced overnight with lentiviral construct luc-19t in the presence of 8 μg/ml polybrene (Sigma-Aldrich). After 24 h, cells were washed three times with sterile filtered PBS, re-suspended in PBS, and re-injected into next generation recipient mice.

At advanced leukemic disease, mice were sacrificed, cells were re-isolated and percentage of PDX AML cells expressing luciferase-CD19t (PDX^luc/19t^) was analyzed on an LSRFortessa Cell Analyzer. PDX^luc/19t^ cells were enriched by magnetic cell separation using CD19 MicroBeads according to the manufacturer’s protocol (Miltenyi Biotech). Enriched cells were re-injected into mice for amplification. Enrichment was repeated once, if necessary, to achieve >90% transgenic cells.

**Generation of knockout cell lines using CRISPR/Cas9 technology**

All gRNAs were designed with the online tool “CHOPCHOP” (http://chopchop.cbu.uib.no/, April 16, 2020) and synthesized as sgRNAs by Thermo Fisher Scientific. All gRNAs were dissolved in an appropriate amount of TRIS buffer (10 mM Tris, pH 8.0) to get working solutions of 100 pmol/µl and aliquoted to avoid freeze-thaw cycles and stored at -20°C until use. To transfect 2 x 10^6^ cells a total of 12,5 µg TrueCut Cas9 Protein v2 (Thermo Fisher Scientific) and 75pmoles gRNA were mixed and incubated for at least 10 min, maximum 30 min at RT to form the CRISPR/RNP complex. Transfection of the RNP was done with the Neon™ Transfection System 100 µl Kit according to the manufacturer’s instructions. Knockout cells were enriched by bulk fluorescence-activated cell sorting (FACS).

**Luciferase-Based Cytotoxicity Assay (LCA)**

Tumor cells were plated in complete RPMI media in white 96-well flat-bottomed plates (Greiner Bio One) with 2 x 10^4^ cells per well. Synthetic D-luciferin (Sigma Aldrich) was added to each well at 4 µg/mL. Effector cells were plated at the indicated effector to target ratio (E:T). E:T titration ranged from 4:1 to 1:32 at therapeutic antibody concentrations of 10 ng/mL. Therapeutic antibody titrations were performed at concentrations from 100 ng/ml in logarithmic steps until 0.1 pg/ml at E:T 1:1 (HL60 and Molm13) and 1:4 (U937). The total volume per well was 200 µL. Plates were incubated in a HERAcell incubator (Heraeus) at 37 °C, 95 % humidity, and 5 % CO2. Bioluminescence was measured using the Tecan SPARK microplate reader (Perkin Elmer) at 37 °C, at the indicated time intervals. Lysis was calculated by the relative luminescence of the testing conditions according to a lysis formula based on a standard dilution series.

**Patient samples for flow cytometry analysis**

BM samples were collected from AML patients at initial-diagnosis or relapse after obtaining written informed consent. This study was approved by the Institutional Ethical Review Board (“Ethikkommission der Medizinischen Fakultät der Eberhard-Karl-Universität und am Universitätsklinikum Tübingen” approval number 819/2017BO1, 674/2017BO2) and performed in accordance with the Declaration of Helsinki.

**Immunophenotyping of tumor cell lines and pediatric AML samples**

Immunophenotyping was performed on a Cytek Aurora (CytekBiosciences) 5-laser spectral flow cytometer. Live/dead dye and antibodies were titrated on cell lines to determine optimal dilution. Cryopreserved patient samples (BM-MNCs) were thawed and treated with DNase 1 in DPBS (pH 7.0-7.2, 0.5 mM MgCl2, 1 mM CaCl2 (Gibco™, Thermo Fisher)) containing 0.5 % (v/v) HSA (supplier) and 20 U/ml DNase 1 (Invitrogen™, Thermo Fisher) prior to staining. Live/dead staining was performed using ZOMBIE UV™ viability dye (BioLegend) at 1:1000 dilution at 4 °C in PBS. To prevent unspecific antibody binding to Fc-receptors, cells were blocked using FcR blocking reagent, human (Miltenyi Biotech). Antibody staining was carried out according to standard operating procedures at 4 ◦C in CliniMACS® PBS/EDTA Buffer supplemented with 0.5 % HSA. Commercial antibodies were purchased from Miltenyi Biotec, BioLegend, and BD Biosciences. The antibody clone and determined optimal dilution regarding supplier recommendation is defined by the clone and dilution factor in brackets. The antigens were CD3 Spark Blue™550 (SK7, 1:5), CD45 BB515 (HI30, 1:25), CD38 BV605 (HB-7, 1:5), CD34 BV711 (8G12, 1:5), CD33 BUV563 (WM53, 1:25), CD135 PE-Cy™5 (BV10A4H2, 1:10), CD371 BUV737 (50C1, 1:25), CD123 APC/Fire™ 810 (6H6, 1:5), IL1RAcP PE-Vio®770 (REA558, 1:5), Mesothelin 1 PE (REA1057, 1:5), CD273 BB700 (MIH18, 1:10), CD274 PE/Fire™ 810 (29E.2A3, 1:10), CD276 APC-Vio®770 (REA1094, 1:25), CD112 PE-Vio®615 (REA1195, 1:5), CD155 AF™700 (SKII.4, 1:25), HLA-DR BV785 (L243, 1:25), and MIC A/B AF™647 (6D4, 1:5).

Single stain reference controls were applied on UltraComp eBeads™ Plus (ThermoFisher) and on cells for antigens listed as follows in appropriate antigen dilution. Molm13 widltype for Viability, CD33, CD123, CD135, IL1RAP, U937 for CD38, CD371, HeLa for MIC A/B, LS for CD276, stem cells for CD34, T cells for CD3, CD45. Samples were acquired and unmixed on Cytek Aurora (CytekBiosciences) 5-laser spectral flow cytometer under “Cytek Assay Settings” in SpectroFlo® V3.0.3 software and further analyzed using FlowJo 10.8 software. Antigen positivity was defined by fluorescence minus one (FMO) controls.

**Flow cytometry-based cytotoxicity assays (antigen evasion)**

To visualize antigen-specific killing, Molm13 wildtype, Molm13 CD33KO, Molm13 CD38KO, and Molm13 CD33/CD38 KO were mixed at a 1:1:1:1 ratio with 1,5 x 10^5^ cells per Molm13 variant (in total 6 x 10^5^ cells) in a FACS tube. AdCARs were added at an E:T ration 1:1. CD33 mAb, CD38 mAb or CD123 mAb and combinations thereof were added at to final concentration of 10 ng/mL as indicated. Tubes were incubated for indicated timepoints in a HERAcell incubator (Heraeus) at 37 °C, 95 % humidity, and 5 % CO2. Analysis was performed on BD FACSCanto™ II flow cytometer. Antibody staining was carried out according to standard operating procedures at 4 ◦C in a CliniMACS® PBS/EDTA Buffer. All commercial antibodies were purchased from (Miltenyi Biotec). The antibody clone is defined by the clone in brackets. The tumor-associated antigens were CD33 VioGreen™ (REA775), CD38 PE-Vio®770 (REA671), CD123 APC-Vio®770 (REA918), CD135 PE (REA786), CD371 VioBlue™ (REA431). CD45 FITC (REA747) and CD3 APC (REA613) were used to differentiate between tumor cells and AdCAR-Ts. 7-AAD was added to exclude dead cells. Samples were acquired on BD FACSCanto™ II flow cytometer in BD FACSDiva™ software and further analyzed using FlowJo 10.8 software. CD33, CD38 and CD123 parameter were included for UMAPs using FlowJo plugin.

**Animals and in vivo model**

For all *in vivo* experiments, 6- to 8-week-old NOD.Cg-PrkdcSCID Il2rgtm1Wjl/SzJ (NSG) mice of female sex from Charles River Laboratories were used and kept in individually ventilated cages (IVC) at a maximum of five animals per cage. General health status of all animals was monitored daily. All experiments were performed according to the guidelines of the Federation of European Laboratory Animal Science Associations (FELASA) in the animal facility of the University clinic of Tübingen. For the U937 *in vivo* studies 1 × 10^6^ U937 cells, stably expressing a firefly luciferase and CD19t gene (luc/19t), were injected into the tail vein on day −4 of the experiment. For Patient derived xenograft (PDX) *in vivo* studies, cells were thawed, washed once in phosphate-buffered saline (PBS), and 1 × 10^6^ cells were injected via the lateral tail vein. Engraftment of the tumor cells was monitored by *in vivo* bioluminescence imaging (BLI) on day 0 and animals were randomized into groups according to tumor burden. All mice received 10 mg human IgG (Gammaguard) by subcutaneous injection twice a week, starting on day 0. LLE-mAB (45 µg) was applied by subcutaneous injection twice per week starting on day 0. 5x10^6^ CAR-T cells were injected intravenously on day 0. Tumor growth was monitored twice per week by BLI. Mice were sacrificed when reaching end point criteria or latest after 50 days according to the approval by the local authorities. For analysis of bone marrow, samples were taken from femur and tibia after mice were sacrificed. Cells were treated with Red Blood Cell (RBC) Lysis Buffer for 10 min and washed in CliniMACS® PBS/EDTA buffer for staining with fluorophore conjugated antibodies against huCD45-FITC, CD3-APC, CD33-Viogreen, CD38-PE-Vio770, CD123-APC-Vio770, CD135-PE, CD371-Vioblue, 7-AAD. Samples were acquired on BD FACSCanto™ II flow cytometer in BD FACSDiva™ software and further analyzed using FlowJo 10.8 software

**Optical Imaging**

For tumor burden monitoring, mice were injected 3 mg D-Luciferin (Perkin Elmer) s.c. for bioluminescence imaging (BLI) following a 5 min uptake period. An IVIS Spectrum Optical Imaging system (Perkin Elmer) was used to acquire BLI scans for 1s, 10s, or 20 s and signals were normalized for the luciferase activity [photons/second]. ROI scans were analyzed using Living Image Software 4 (Perkin Elmer), by drawing regions of interest (ROI) around the whole animal.

**Data visualization**

Data was visualized using Prism 9 version 9.2.0 (283) (GraphPad Software Inc.). Figures were generated using the vector graphics software Inkscape 1.2.2 (732a01da63, 2022-12-09).

Supplementary Table 2: Media and buffers:

| **Name** | **Final composition** |  | **Usage** |
| --- | --- | --- | --- |
| CliniMACS/HSA buffer | 500 ml CliniMACS® PBS/EDTA Buffer  + 0.5% HSA |  | Staining for flow  cytometry |
| DNase treatment buffer | DPBS (pH 7.0-7.2, 0.5 mM MgCl_2_, 1 mM CaCl_2_)  + 0.5% HSA  + 20 U/ml DNase 1 |  | Thawing primary cells |
| DMEM 10% | 500 ml DMEM medium + 50 ml FCS (10%)  + 5 ml Penicillin-Streptomycin (1%)  + 5 ml L-Glutamine (1%) |  | Cultivation of cell lines |
| Freezing medium | 90% FCS/HSA  + 10% DMSO |  | Freezing of cell lines, respectively primary cells |
| RPMI 10% | 500 ml RPMI 1640 medium  + 50 ml FCS  + 5 ml Penicillin-Streptomycin (1%)  + 5 ml L-Glutamine (1%) |  | Cultivation of cell lines |
| TexMACS  culture medium | TexMACS medium 500 ml  + 5 ml Penicillin-Streptomycin  + 100 µl IL-7 (10 ng/ml)  + 100 µl IL-15 (5 ng/ml) |  | Cultivation of primary T cells |
| TexMACS  thawing medium | TexMACS medium 500 ml  + 50 ml FCS  + 5 ml Penicillin-Streptomycin |  | Thawing primary T cells |
| PDX Medium | 500 ml RPMI 1640 medium  + 100 ml FCS  + 5 ml Penicillin-Streptomycin (1%)  + 5 ml L-Glutamine (1%)  + 0.6% mixture of rh insulin/human transferrin/sodium selenite |  | Overnight transduction of primary AML |
|  | + 1 mM sodium pyruvate  + 50 μM 1-thioglycerole |  |  |
|  |  |  |  |

Supplementary Table 3: Cells

| **Cell line** |  | **Specification** |  | **Details** |
| --- | --- | --- | --- | --- |
| Molm13 |  | Human AML-derived cell line |  | In suspension |
| U937 |  | Human histiocytic lymphoma-derived monocyte-like cell line |  | In suspension |
| HL60 |  | Human AML-derived cell line |  | In suspension |
| PBMC |  | Human, isolated from whole blood from healthy donors |  | In suspension |
| Stem cells |  | Human, isolated from bone marrow of healthy donors |  | In suspension |
| T cells |  | Human, isolated from whole blood from healthy donors |  | In suspension |

Supplementary Table 4: Antibodies for flow-cytometry

| **Antigen** | **Conjugate** | **Clone** | **Supplier** | **Isotype** | **Cat. #** |
| --- | --- | --- | --- | --- | --- |
| CD3 | Spark Blue™550 | SK7 | BioLegend® | Mouse IgG1, κ | 344851 |
| CD3 | APC | REA613 | Miltenyi Biotec | recombinant  human IgG1 | 130-113-135 |
| CD33 | BUV563 | WM53 | BD Biosciences | Mouse BALB/c  IgG1, κ | 741369 |
| CD33 | VioGreen™ | REA775 | Miltenyi Biotec | recombinant  human IgG1 | 130-111-025 |
| CD34 | BV711 | 8G12 | BioLegend® | Mouse IgG1, κ | 745543 |
| CD38 | BV605 | HB-7 | BioLegend® | Mouse IgG1k | 356642 |
| CD38 | PE-Vio®770 | REA671 | Miltenyi Biotec | Recombinant  human IgG1 | 130-113-432 |
| CD45hu | BB515 | HI30 | BD Biosciences | Mouse IgG1, κ | 564586 |
| CD45hu | FITC | REA747 | Miltenyi Biotec | Recombinant  human IgG1 | 130-110-631 |
| CD45mouse | Pe-Vio 770 | REA737 | Miltenyi Biotec | Recombinant  human IgG1 | 130-110-799 |
| CD112 | PE-Vio®615 | REA1195 | Miltenyi Biotec | Recombinant  human IgG1 | 130-122-784 |
| CD123 | APC-Fire™ 810 | 6H6 | BioLegend® | Mouse IgG1, κ | 306053 |
| CD123 | APC-Vio®770 | REA918 | Miltenyi Biotec | Recombinant  human IgG1 | 130-115-267 |
| CD135 | PE-Cy™5 | BV10A4H2 | BioLegend® | Mouse IgG1k | 313310 |
| CD135 | PE | REA786 | Miltenyi Biotec | Recombinant  human IgG1 | 130-111-587 |
| CD371 | BUV737 | 50C1 | BD Biosciences | Mouse IgG2a, κ | 748888 |
| CD371 | VioBlue | REA431 | Miltenyi Biotec | Recombinant  human IgG1 | 130-123-533 |
| IL1RAP | PE-Vio®770 | REA558 | Miltenyi Biotec | Recombinant  human IgG1 | 130-108-754 |
| MSLN | PE | REA1057 | Miltenyi Biotec | Recombinant  human IgG1 | 130-118-095 |
| MIC A/B | AF™647 | 6D4 | BioLegend® | Mouse IgG2a, κ | 320914 |

Supplementary Table 5: Single guide RNAs

| Target | Internal no. | Sequence 5`🡪 3` | Supplier |
| --- | --- | --- | --- |
| CD33 | 64 | GAGTCAGTGACGGTACAGGA | Invitrogen™ TrueGuide™ |
| CD38 | 34 | GATCCTCGTCGTGGTGCTCG | Invitrogen™ TrueGuide™ |

Supplementary Table 6: Reagents and Kits

| Product | Supplier | Cat. # |
| --- | --- | --- |
| 7-AAD | BD Pharmingen™ | 559925 |
| AdCAR detection reagent  PE and Biotin tag | Miltenyi Biotec | - |
| Aquaresist | VWR International | 462-7000 |
| BD FACSClean | Becton, Dickinson and  Company | 340345 |
| BD FACSFlow | Becton, Dickinson and  Company | 342003 |
| BD FACS Shutdown Solution | Becton, Dickinson and  Company | 34224 |
| Biocoll® separating solution,  density 1.077 g/ml | Bio&SELL | L6115 |
| Brilliant Stain Buffer, 5 ml | BD Biosciences | 563794 |
| Cas9-NLS | UC Berkeley QB3 Macrolab | - |
| CD4 MicroBeads | Miltenyi Biotec | 130-045-101 |
| CD8 MicroBeads | Miltenyi Biotec | 130-045-201 |
| CD19 MicroBeads, human | Miltenyi Biotec | 130-050-301 |
| CD45 Microbeads, human | Miltenyi Biotec | 130-045-801 |
| CliniMACS® PBS/EDTA buffer | Miltenyi Biotec | 200-070-025 |
| Compensation Beads  UltraComp eBeads™ Plus | ThermoFisher | 01-3333-42 |
| CryoSure-DMSO | WAK-Chemie Medical | WAK-DMSO-70 |
| Descosept AF | Dr. Schumacher GmbH | 00-311-010 |
| D-Luciferin | Sigma-Aldrich | L9504 |
| DMEM culture medium | Gibco™, Thermo Fisher | 41965039 |
| Dnase 1  20.000 units | Invitrogen™,  Thermo Fisher Scientific | 18047019 |
| DPBS, magnesium, calcium | Gibco™, Thermo Fisher | 14040-117 |
| HSA, 20% | Takeda | 0749161 |
| Heparin-Natrium  25.000 I.E./5 ml | Leo Pharma | 15261203 |
| Human IL7, premium grade | Miltenyi Biotec | 130-095-362 |
| Human IL15, premium grade | Miltenyi Biotec | 130-095-765 |
| Insulin-Transferrin-Selenium (ITS -G) (100X) | Gibco | 41400045 |
| Ethanol denatured | SAV Liquid production | ETO-10000-99-1 |
| FCS | Gibco™, Thermo Fisher | 10270-106 |
| FcR Blocking Reagent | Miltenyi Biotec | 130-059-901 |
| L-glutamine 200 mM | Sigma-Aldrich | G7513 |
| MACS® Comp Bead Kit,  anti-REA | Miltenyi Biotec | 130-104-693 |
| Neon™ Transfection System 100 µL Kit | Invitrogen | MPK10096 |
| PBS, sterile | Sigma-Aldrich | D8537 |
| Penicillin (10.000 U/ml)-Streptomycin (10 mg/ml) | Sigma-Aldrich | P0781 |
| Sodium pyruvate (100 mM), 100X | Gibco | 11360-70 |
| RPMI 1640 medium | Sigma-Aldrich | R0883 |
| SpectroFlo® QC Beads | Cytek® Biosciences | SKU B7-10001 |
| TexMACS media | Miltenyi Biotec | 130-097-196 |
| 1-thioglycerole | Sigma-Aldrich | M6145 |
| Trypsin-EDTA solution | Sigma-Aldrich | T3924 |
| T Cell TransAct™ | Miltenyi Biotec | 130-111-160 |
| Zombie UV™ Viability Dye | BioLegend® | 423107 |

**Supplementary Table / Figure Legends**

**Supplementary Table 1: Patient Characteristics.**

Characteristics of analyzed AML patients. From left to right: Patient ID, sex, age at primary diagnosis, primary or relapse at sample collection, AML type (FAB classification), genetic.

**Supplementary Figure 1: Gating strategy of bone marrow analysis, expression analysis of AdCAR T cells, and CD33/CD38 expression on activated T cells.**

**A** Flow cytometry gating strategy of bone marrow from an AML patient. From left to right: lymphocytes defined via FSC-A and SSC-A, doublet discrimination via FSC-A vs. FSC-H and SSC-A vs. SSC-H, dead cells discrimination via ZOMBIE UV™ viability dye, AML blasts defined as CD45 dim. **B** Gating strategy of healthy bone marrow: From left to right: lymphocytes defined via FSC-A and SSC-A, doublet discrimination via FSC-A vs. FSC-H and SSC-A vs. SSC-H, dead cells discrimination via ZOMBIE UV™ viability dye, CD45 dim defining HSPCs as CD34^+^/CD38^+^ and HSCs as CD34 CD34^+^/CD38^-^. **C** Exemplary AdCAR T expression analysis determined by flow cytometry and stained with AdCAR detection reagent PE (Miltenyi Biotec).  **D** CD33 and CD38 expression on activated T cells is shown as histograms of an exemplary donor (left). CD33 and CD38 expression on activated T cells from 5 different donors (right). Each data point represents an individual heathy donor, horizontal lines the mean value ± standard deviation (SD).

**Supplementary Figure 2: Flow chart of LLE-mAb production.**

VH and LH sequences for the respective mAbs were cloned on an Fc-attenuated (L234A/L235A (LALA)) IgG1 backbone in a pcDNA™3.1 (+) mammalian expression vector. ExpiCHO™ cells were cultured until reaching the desired cell amount a density for transient transfection. Transfection was performed according to the manufacturer’s instructions, using the high titer protocol. Antibodies in the clarified and filtered supernatant were harvested by centrifugation and purified using rProtein A GraviTrap columns (Cytiva) followed by desalting with PD10 Sephadex G25 columns (Cytiva). Biotin conjugation of purified antibodies was performed using 3-fold molar excess of biotin-LC-LC-NHS (Thermo Fisher), followed by separation of the antibody/label mix again on a Sephadex G25 column (Cytiva). Successful conjugation was confirmed by flow cytometry on cell lines expressing the target antigen and secondary staining with a fluorophore-conjugated anti-biotin antibody (Miltenyi Biotec) and functional assays. Purity was analyzed by SDS Gel analysis.

**Supplementary Figure 3: Flow cytometry analysis of healthy bone marrow.**

A UMAP based on the expression of CD45, CD3, CD34, CD33, CD38, CD123, CD135 and CD371 as well as FSC and SSC signals, as determined by flow cytometry, of an exemplary healthy bone marrow sample. B Antigen expression of CD45, CD3, CD34, CD33, CD38, CD123, CD135 and CD371, each dot representing one cells. A Clustering compared with antigen expression allows a clear separation of the main cell populations T cells, Monocytes, not T lymphocytes, as well as HSCs, and HSPCs.

**Supplementary Figure 4: In vitro evaluation of multiplex targeting by AdCAR-T vs. U937.**

U937 wildtype (WT), U937 CD33KO, U937 CD38KO, and U937 CD33/CD38 KO were mixed at a 1:1:1:1 ratio. AdCAR-T were added at an E:T ratio of 1:1. LLE-aCD33, LLE-aCD38, or LLE-aCD371 as well as combinations thereof were added to reach a final concentration of 10 ng/mL. B UMAP, representing batched surviving target cells after 48h of incubation of all conditions (n=3 each condition), based on the expression of CD33, CD38 and CD371, as determined by flow cytometry. From left to right, viable target cells for indicated conditions are plotted in color. From top to bottom, expression of CD33, CD38, and CD371 are color-coded.

**Supplementary Figure 5: Bone marrow analysis of PDX mice.**

A Gating strategy of bone marrow end point analysis from the in vivo PDX experiment. Lymphocytes defined via FSC-A and SSC-A, doublet discrimination via FSC-A vs. FSC-H and SSC-A vs. SSC-H, dead cells discrimination via 7AAD. Tumor cells are defined as CD45 human+ / CD3-. B Expression profile of CD33, CD38, CD123, CD135, and CD371 of the PDX cells from the untreated control group (Tumor only). C CD3+ frequency in bone marrow at end point analysis. Each group represents 4-5 mice, horizontal lines the mean value ± standard deviation (SD). Statistical analysis was performed using ordinary one-way ANOVA and Tukey’s multiple comparison test. ns, not significant. *, p ≤ 0.0332 **, p ≤ 0.021. ***, p ≤ 0.002. ****, p ≤ 0.0001. The full table of the respective statistical analysis is provided in the ***Additional file 1*.**

**Supplementary Figure 6:** **In vivo validation of multiplex targeting by AdCAR-T cells in a second PDX model.**

**A** Schematic depiction of the in vivo experiment: NSG mice were engrafted with 1x10^6^ PDX ^luc/CD19t^ cells on day -7 via tail vein injection (i.v.). A total of 5x10^6^ AdCAR-T cells were injected i.v. on day 0. A total of 45 µg of the indicated AM (LLE-aCD33, LLE-aCD38, or LLE-aCD371) or a combination thereof was injected subcutaneously (s.c.) twice a week starting on day 0. Untreated mice (tumor only) and mice injected with PBS instead of AM (AdCAR-T only) served as negative controls (n=3 per group, n=5 in multiplex group). Tumor load was monitored by BLI. Mice were sacrificed when they reached the endpoint criteria. **B** UMAP based on the expression of CD45, CD33, CD38, CD123, CD135 and CD371 as well as FSC and SSC signals, as determined by flow cytometry, of PDX cells prior to injection, expression of CD33, CD38, CD123, CD135 and CD371 on AML blasts **C** BLI images at the indicated time points (exposure time 10 sec.). **D** Total flux [photons/second] of in vivo bioluminescence blotted over time for individual animals. **E** Kaplan‒Meier curves for reaching endpoint criteria. Statistical analysis was performed using two-way ANOVA and Tukey’s multiple comparison test. ns, not significant. *, p ≤ 0.0332 **, p ≤ 0.021. ***, p ≤ 0.002. ****, p ≤ 0.0001. The full table of the respective statistical analysis is provided in **Additional file 1.**

**Supplementary Table 1**

| **ID** | **Sex** | **Age  [Years]** | **Primary/  Relapse** | **AML Type** | **Genetic** | **Time to Relapse  [Year]** | **Dead/Alive** |
| --- | --- | --- | --- | --- | --- | --- | --- |
| P1 | m | 1,9 | Primary | M1 | Monosomie 7 | 2,4 | Alive |
| P2 | m | 14,1 | Primary | M2 | N/A | - | Alive |
| P3 | f | 9,8 | Primary | M2 | AML1-ETO-fusion | 1,1 | Dead |
| P4 | m | 15,3 | Primary | M2 | AML1-ETO-fusion | - | Alive |
| P5 | f | 14,7 | Primary | M2 | N/A | - | Alive |
| P6 | f | 13,5 | Primary | M2 | N/A | - | Alive |
| P7 | m | 0,6 | Primary | M4 | t(X;11) not involoving KMT2A | 0,1 | Alive |
| P8 | m | 9,9 | Primary | M4 | NPM1 mutation, FLT3-TLD mutation | - | Alive |
| P9 | m | 17,7 | Primary | M4 | CBFB-MYH11-fusion | 0,6 | Alive |
| P10 | f | 6,8 | Primary | M5 | TPMT-Mutation | - | Alive |
| P11 | f | 17,7 | Primary | M5 | N/A | - | Alive |
| P12 | m | 7,7 | Primary | M5 | Deletion 5q | 1,1 | Alive |
| P13 | m | 3,3 | Primary | M5 | MLL-rearrangement not further defind | - | Alive |
| P14 | f | 1,7 | Primary | M7 | N/A | - | Alive |
| P15 | m | 14,3 | Primary | MDS RAEB I | WT1 mutation, AXSL1 mutation, FLT3-TKD mutation, GATA2 mutation | 3,5 | Alive |
| P16 | m | 11,6 | Primary | MDS-EB-t | Trisomie 8 | - | Alive |
| P17 | m | 12,9 | Primary | - | N/A | 3,0 | Alive |
| P18 | m | 10,5 | Primary | - | N/A | 0,4 | Dead |
| P19 | m |  | Primary | - | N/A | - | Alive |
| P20 | m | - | Primary | - | N/A | - | Alive |
| P21 | f | 13,0 | Relapse | M2 | WT1 mutation, FLT3-TLD mutation | 0,8 | Dead |
| P22 | m | 10,0 | Relapse | M2 | N/A | 0,5 | Dead |
| P23 | f | 13,8 | Relapse | M2 | AML1-ETO-fusion | 0,4 | Dead |
| P24 | f | 12,9 | Relapse | M2 | N/A | 1,7 | Alive |
| P25 | m | 1,3 | Relapse | M3 | PML-RARa-fusion negativ | 7,0 | Alive |
| P26 | m | 15,1 | Relapse | M4 | N/A | 0,9 | Alive |
| P27 | m | 4,1 | Relapse | M4 | AML1-ETO-fusion | 1,6 | Alive |
| P28 | f | 5,4 | Relapse | AML M5 | N/A | 3,2 | Alive |
| P29 | f | 14,8 | Relapse | - | N/A | 0,2 | Dead |

**Supplementary Figure 1**


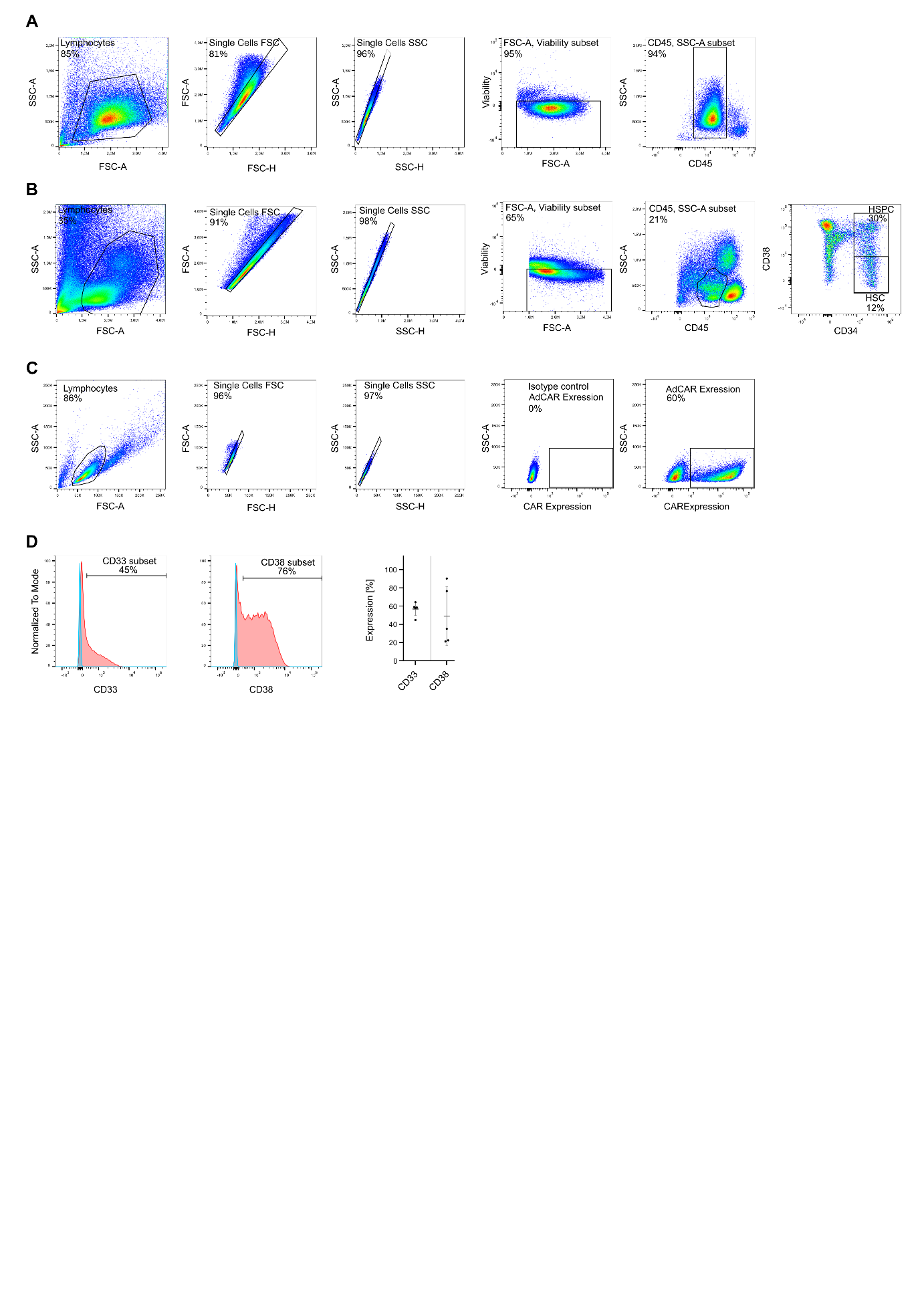


**Supplementary Figure 2**


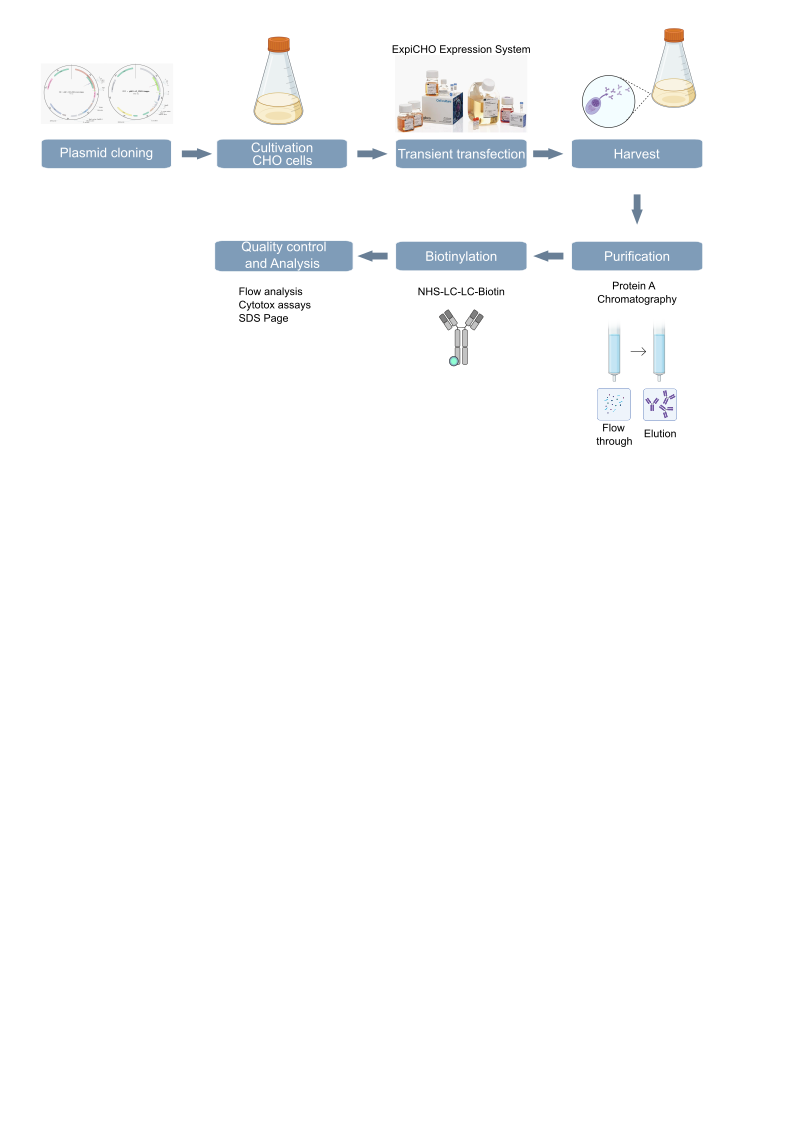


**Supplementary Figure 3**


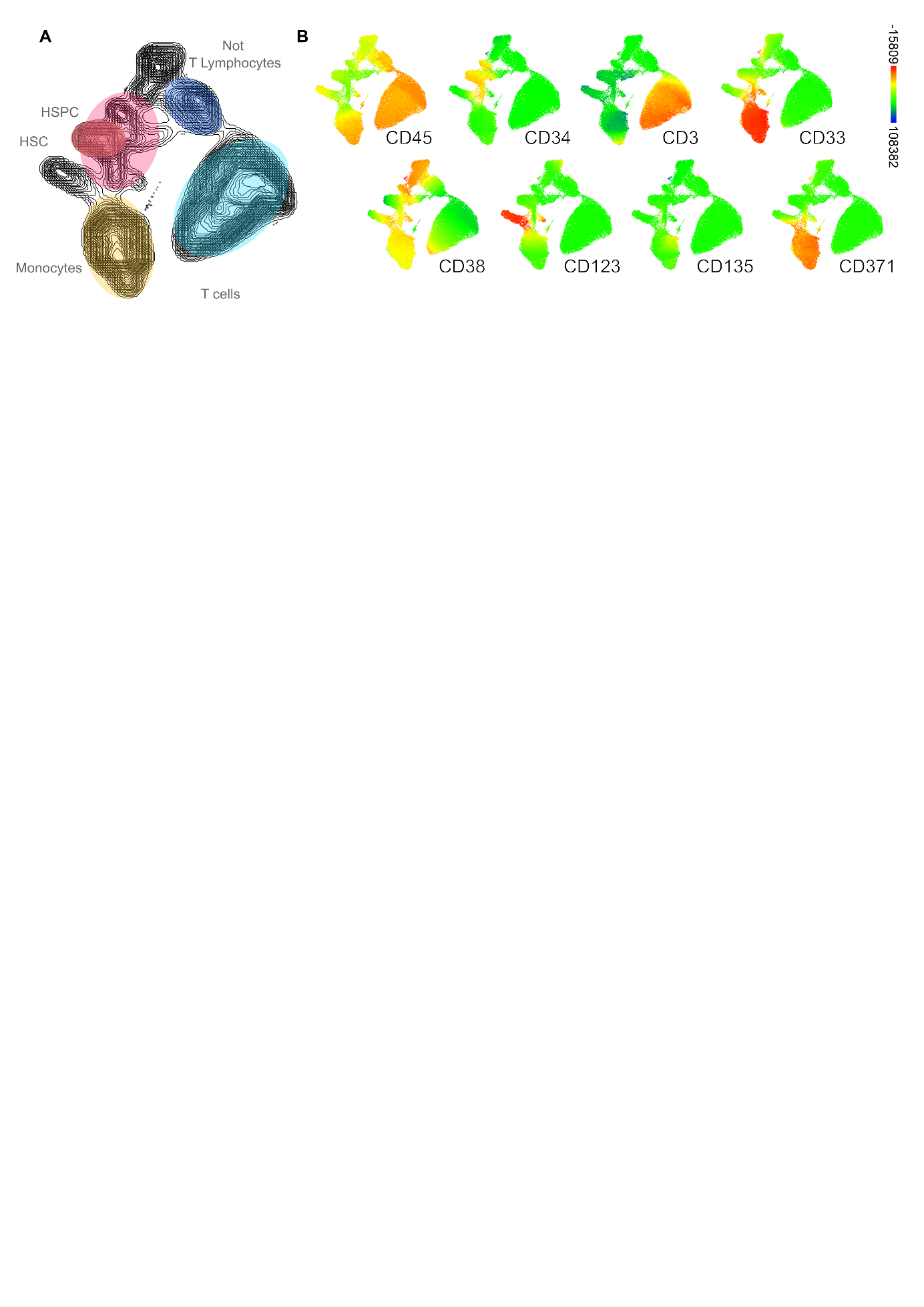


**Supplementary Figure 4**


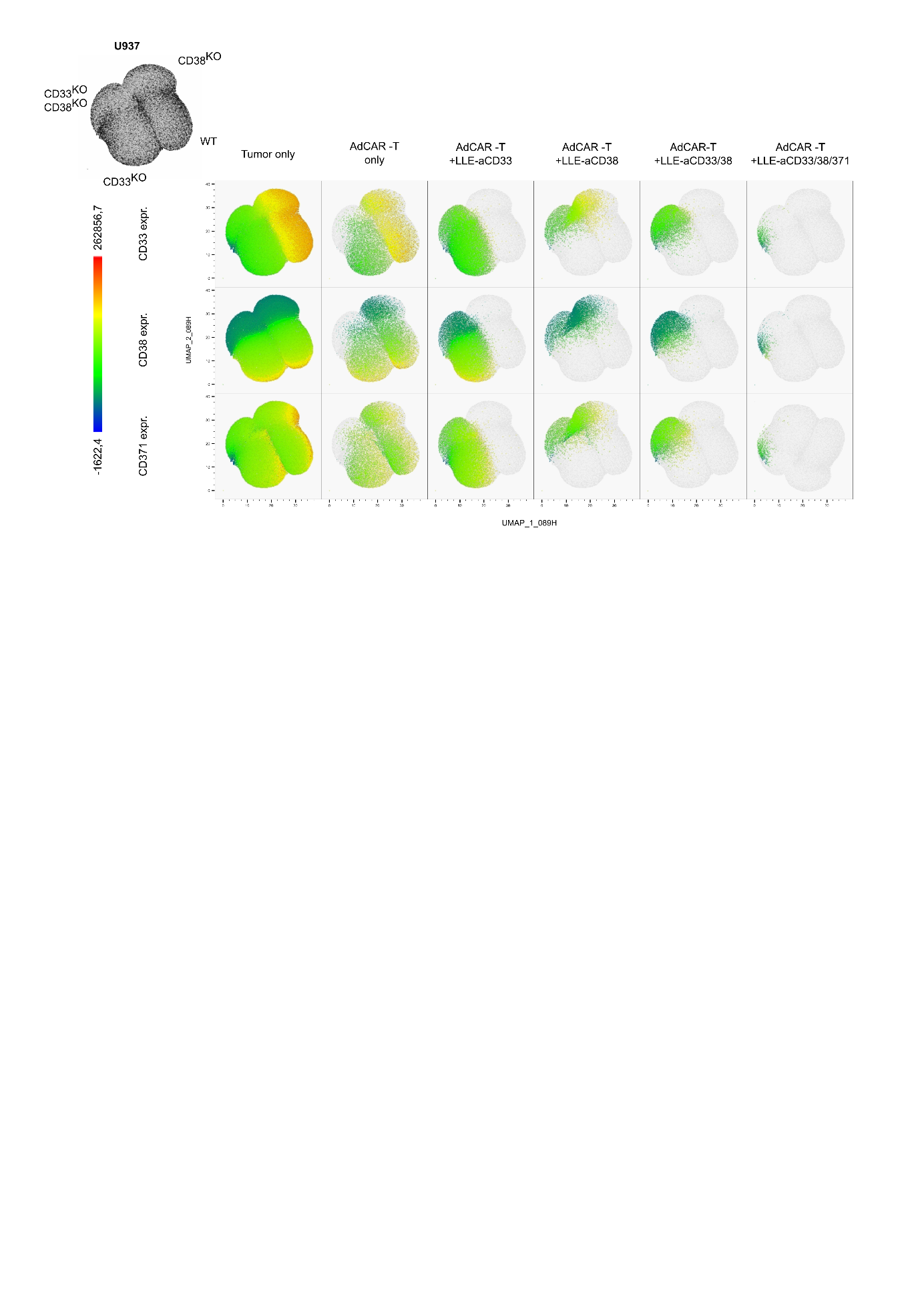


**Supplementary Figure 5**


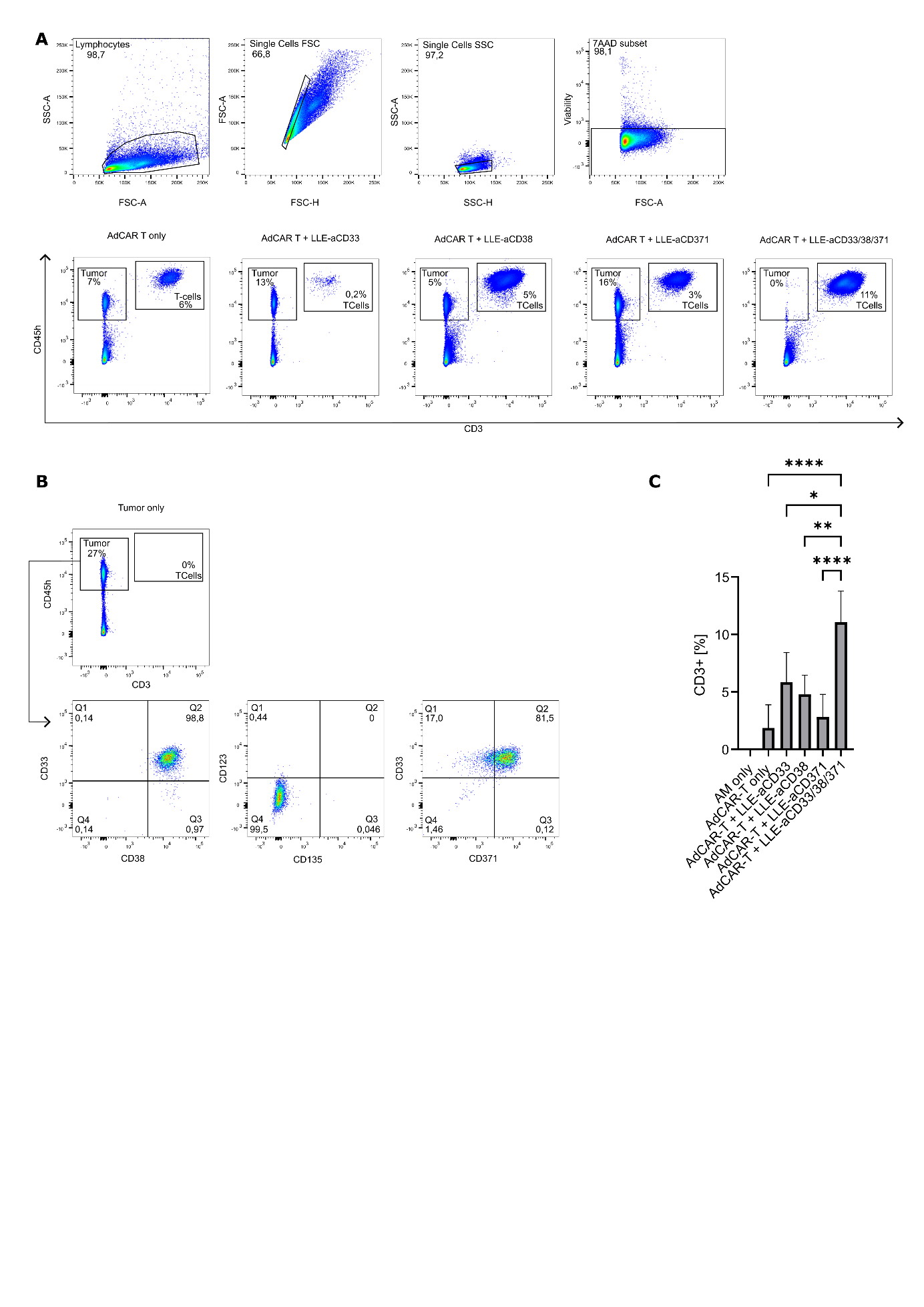


**Supplementary Figure 6**
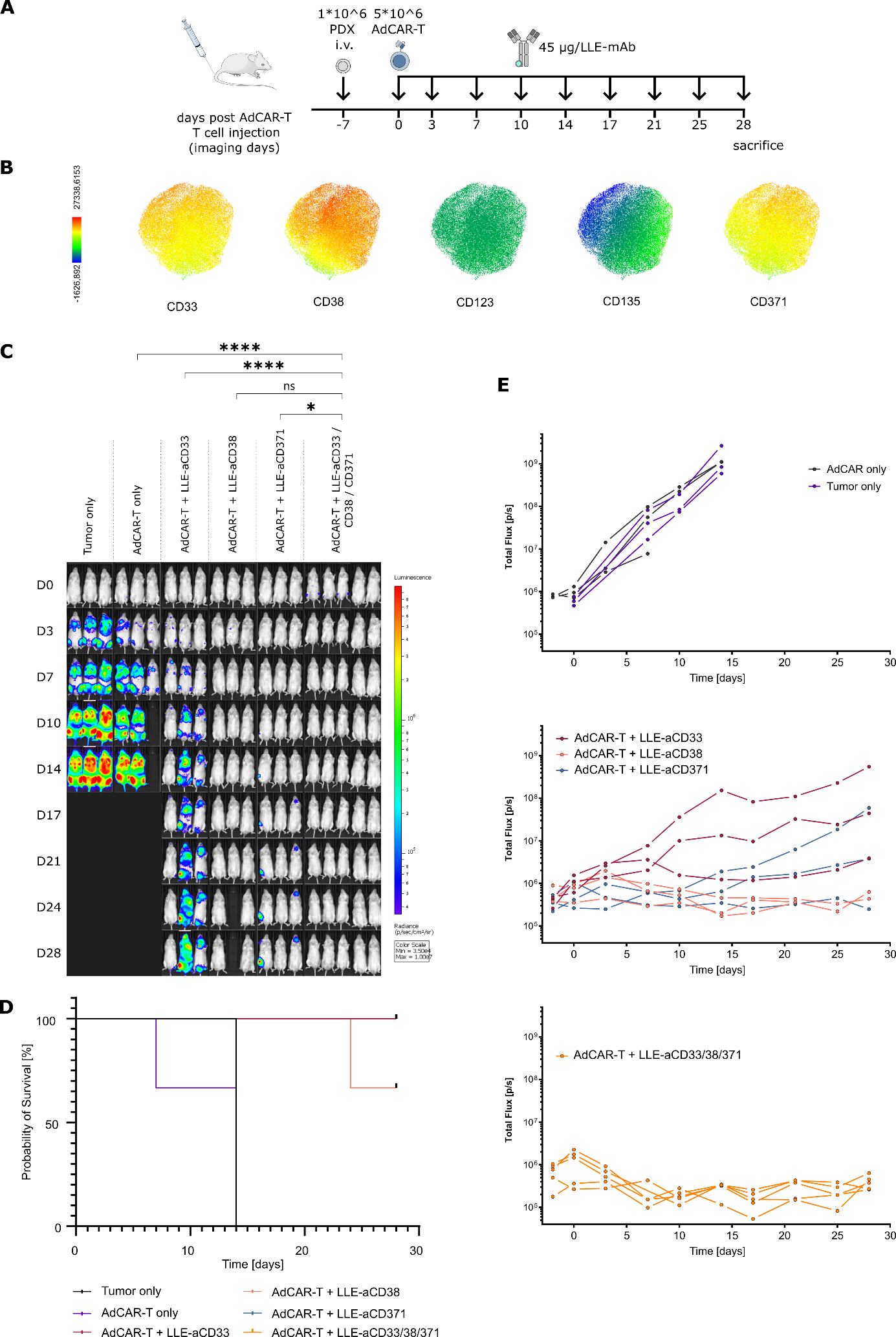

Supplement: Supplementary file 1 — Supplemental Materials [file 41375_2024_2351_MOESM1_ESM.docx]
